# Simulating Assembly Landscapes for Comprehensive Understanding of Supramolecular Polymer–Solvent Systems

**DOI:** 10.1021/jacs.2c12941

**Published:** 2023-02-09

**Authors:** Stef A. H. Jansen, Elisabeth Weyandt, Tsubasa Aoki, Takayoshi Akiyama, Yoshimitsu Itoh, Ghislaine Vantomme, Takuzo Aida, E. W. Meijer

**Affiliations:** † Institute for Complex Molecular Systems, Eindhoven University of Technology, P.O. Box 513, 5600 MB Eindhoven, The Netherlands; ‡ Laboratory of Macromolecular and Organic Chemistry, Eindhoven University of Technology, P.O. Box 513, 5600 MB Eindhoven, The Netherlands; § Department of Chemistry and Biotechnology, School of Engineering, The University of Tokyo, 7-3-1 Hongo, Bunkyo-ku, Tokyo 113-8656, Japan; ∥ RIKEN Center for Emergent Matter Science, 2-1 Hirosawa, Wako, Saitama 351-0198, Japan; ⊥ School of Chemistry and RNA Institute, UNSW, Sydney, NSW 2052, Australia

## Abstract

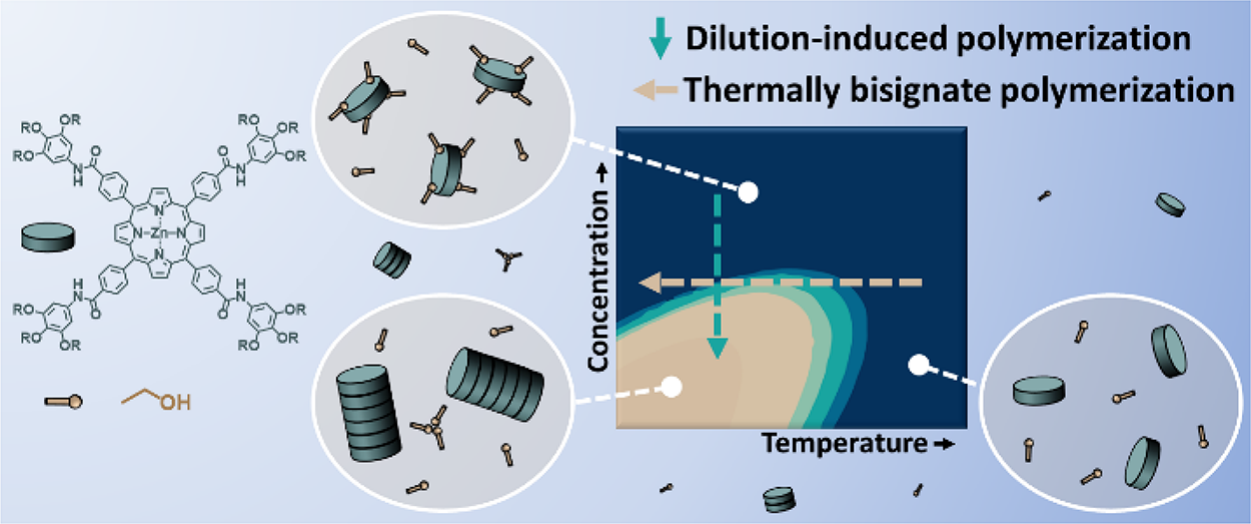

Complexity in supramolecular polymer systems arises from interactions between different components, including solvent molecules. By varying their concentration or temperature in such multicomponent systems, complex phenomena can occur such as thermally bisignate and dilution-induced assembly of supramolecular polymers. Herein, we demonstrate that both these phenomena emerge from the same underlying interaction mechanism between the components. As a model system, amide-decorated supramolecular polymers of porphyrins were investigated in combination with aliphatic alcohols as hydrogen-bond scavengers, and thermodynamic mass-balance models were applied to map the three-dimensional assembly landscapes. These studies unveiled that the interaction between hydrogen-bond scavengers and monomers is temperature-dependent and becomes dominant at high monomer concentrations. With these insights, we could exploit competitive monomer–alcohol interactions to prompt the dilution-induced assembly of various common monomers as well as bisignate assembly events. Moreover, kinetic insights were obtained by navigating through the assembly landscape. Similar to phase diagrams of covalent polymers, these assembly landscapes provide a comprehensive picture of supramolecular polymerizations, which helps to precisely regulate the system properties. The generality of this approach using assembly landscapes makes it relevant for any supramolecular system, and this enhanced control will open the door to build complex and functional supramolecular polymer systems.

## Introduction

Supramolecular polymers^1,2^ are promising functional materials for biomedical^3−5^ and optoelectronic^6−10^ applications because of their adaptive and highly ordered nature.^11−13^ Novel functionalities in supramolecular polymers are often achieved by combining multiple assembling components into supramolecular polymer systems.^14,15^ Despite the recent progress made to elucidate pathway complexity^16^ in these systems, competitive interactions between different components lead to complex assembly properties that are difficult to predict and control.^17,18^ Recently, special attention has been devoted to the role of solvents as additives in supramolecular systems as they are widely accessible and often decisive for the assembly properties.^19−21^ Our groups have reported two new concepts of counterintuitive assembly phenomena caused by competitive solvent–solute interactions. Because of these competitive interactions, supramolecular polymerization could be prompted by diluting^22−24^ or heating^25^ the system, which never happens in ordinary supramolecular assemblies. These reports suggest that solvent–solute interactions could be a useful strategy to gain fine control over supramolecular polymers and build complex structures.

Our first report on the dilution-induced supramolecular polymerization of porphyrins uses small amounts of pyridine mixed with a Zn-centered porphyrin monomer that normally forms stable supramolecular polymers.^22^ The pyridine coordinates to the zinc in the porphyrin specifically at high concentrations, sequestering Zn-porphyrin monomers into pyridine–Zn-porphyrin complexes. By diluting the sample, the equilibria of the competing interactions shift, and the supramolecular polymerization occurs. Similarly, in the thermally bisignate supramolecular polymerization, the free monomers are stabilized by hydrogen bonding of alcohol additives to the monomers.^25^ This stabilization diminishes at high temperatures, allowing the formation of supramolecular polymers. Distinctively, at low temperatures, the alcohol additive forms nanoclusters and therefore less effectively sequestrates the monomers, leading to the formation of supramolecular polymers as well. Thus, the competitive interactions between the amides of the monomers and additives are delicately balanced to stabilize the free monomers mainly at intermediate temperatures, yielding supramolecular polymers either by heating or cooling.^26^ For both these concepts, detailed studies unveiled the competitive interactions that caused the unconventional polymerization events. However, it remains unclear how these concepts relate to each other and how supramolecular systems can be formulated to display these specific assembly properties under the desired conditions.

**Figure 1:**
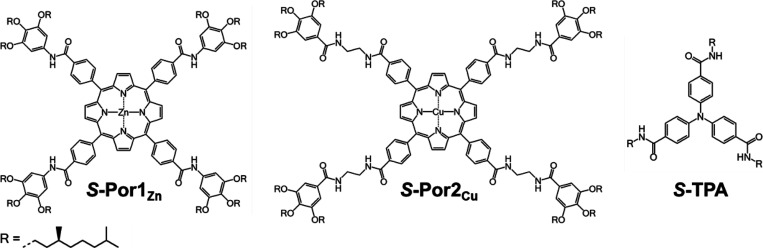
Molecular structures of supramolecular monomers ***S*-Por1_Zn,_**(22,33)***S*-Por2_Cu_**,^25^ and ***S*-TPA**.^34^

In covalent polymer science, many similar fundamental challenges have been addressed to enable the extensive innovations that the field has contributed to.^27,28^ In this regard, the conception of phase diagrams has been helpful in elucidating the intricacies of polymer phase behavior.^29,30^ Here, we aim to translate this methodology to supramolecular polymer–solvent systems involving alcohol additives using several monomers depicted in Figure 1. By utilizing computational analyses^26,31,32^ in combination with spectroscopic techniques, assembly landscapes of the supramolecular polymers could be constructed. We demonstrate that these assembly landscapes provide a comprehensive picture of the assembly properties in supramolecular polymer–solvent systems. Simulation of assembly landscapes following this general approach can be readily extended to different supramolecular systems, which will aid the development of new methodical approaches to build functional supramolecular systems.

## Results and Discussion

### Monomer Solvation by Alcohols

**Figure 2:**
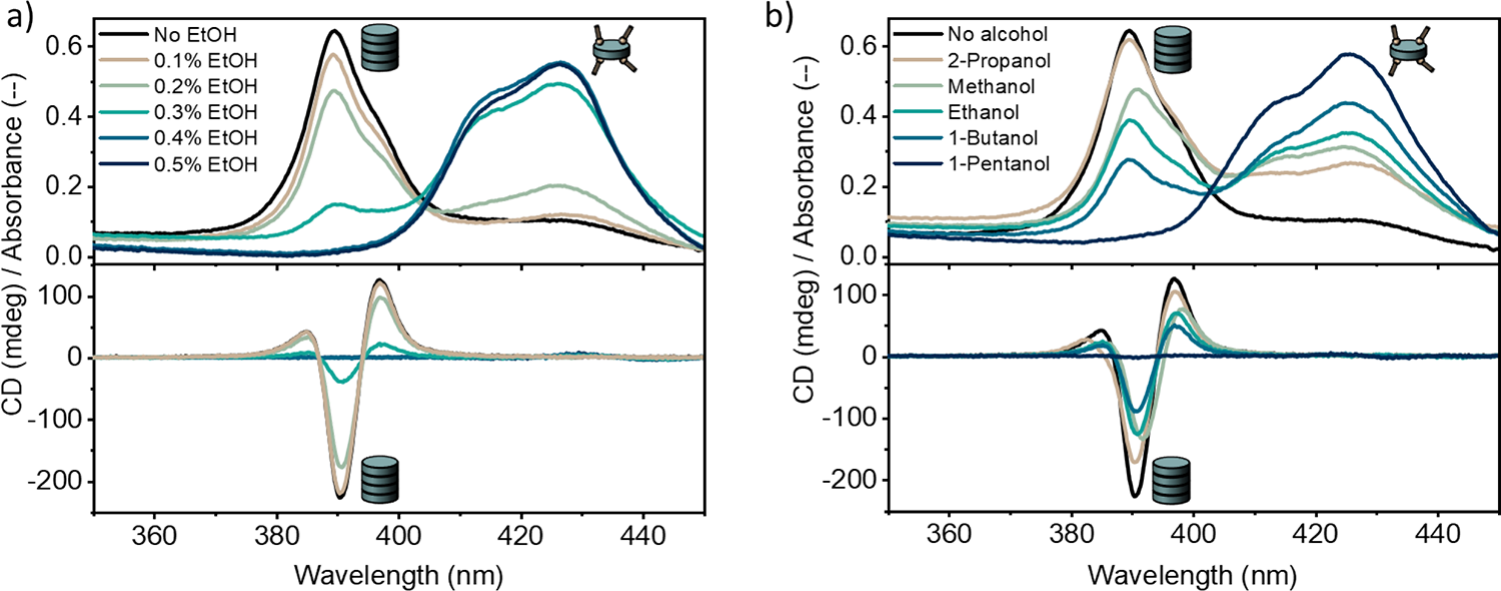
(a) Absorption (top) and ECD (bottom) spectra of 20 μM ***S*-Por1_Zn_** in MCH with 0 (black) to 0.5 (dark blue) vol% (0–4280 equiv) EtOH at 20 °C; (b) absorption (top) and ECD (bottom) spectra of 20 μM ***S*-Por1_Zn_** in MCH with 2570 equiv of various aliphatic alcohols at 20 °C. Small amounts of aliphatic alcohol can solvate ***S*-Por1_Zn_** and lead to disassembly of its supramolecular polymers.

The supramolecular polymerization of the enantiopure porphyrin derivative ***S*-Por1_Zn_** (Figure 1) in methylcyclohexane (MCH) has previously been reported in detail.^35^ The formation of helical H-type supramolecular polymers can be monitored by absorption and electronic circular dichroism (ECD) spectroscopy as these assemblies show a distinct absorption maximum at 391 nm, which is blue-shifted in comparison to the absorption of the free monomer at 421 nm. As a next step, the stability of ***S*-Por1_Zn_** helical supramolecular polymers in the presence of hydrogen-bond scavengers was assessed by the addition of ethanol to 20 μM solutions of ***S*-Por1_Zn_** in MCH at 20 °C. ***S*-Por1_Zn_** was fully disassembled when a minimum of 0.4 vol % (3425 equiv) ethanol was added (Figure 2a). Other aliphatic alcohols showed the same effect on ***S*-Por1_Zn_** in MCH, although the binding affinity for ***S*-Por1_Zn_** was different for each alcohol (Figure 2b). Thus, analogous to previous reports,^25,26,36^ small amounts of alcohols can solvate the monomers, thereby causing the disassembly of hydrogen-bonded supramolecular polymers.

**Figure 3:**
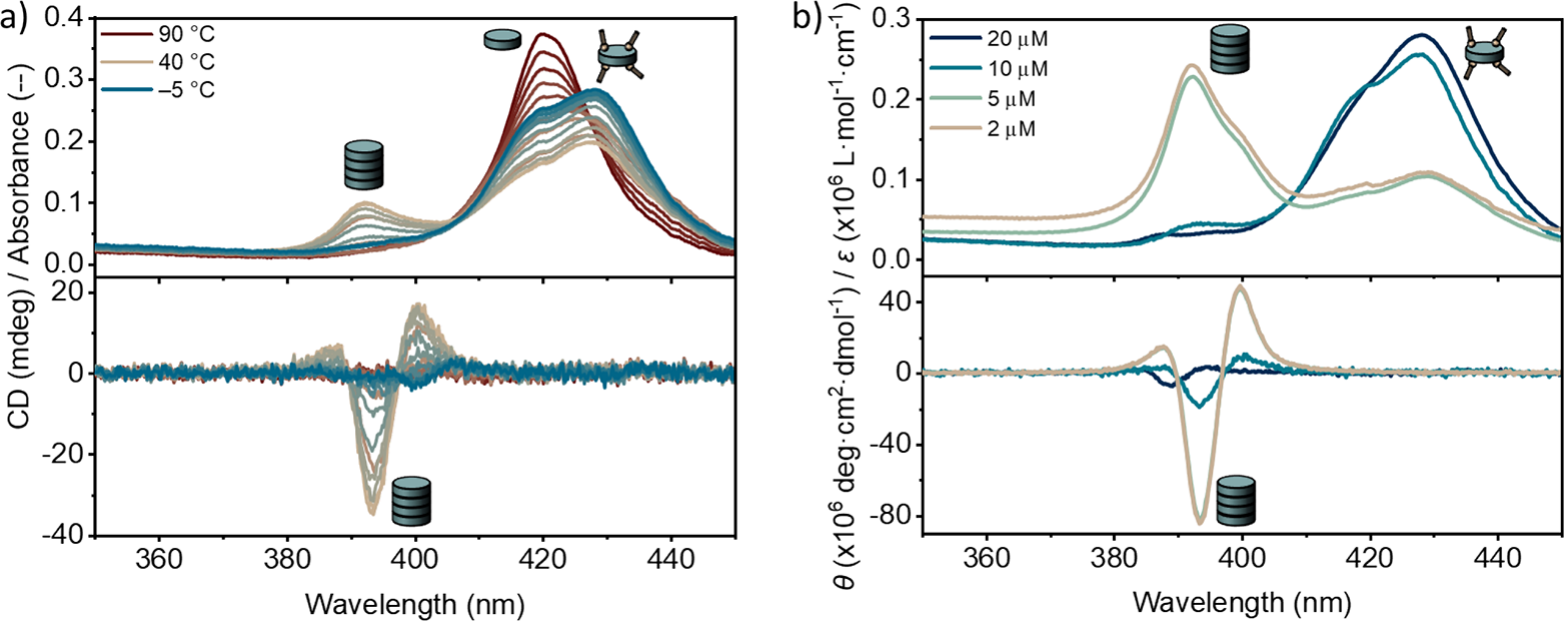
(a) VT-absorption (top) and VT-ECD (bottom) spectra of 10 μM ***S*-Por1_Zn_** in MCH with 4280 equiv of EtOH at a 1 K/min cooling rate. A temperature range was observed where supramolecular polymerization occurred. (b) Absorption (top) and ECD (bottom) spectra of ***S*-Por1_Zn_** in MCH with 4280 equiv of EtOH at 20 °C. ***S*-Por1_Zn_** showed dilution-induced supramolecular polymerization.

In previous reports, sequestration of monomers caused intricate assembly phenomena due to competing interactions within the system.^22,25^ Therefore, we anticipate complex assembly properties emerging as a result of the interaction between alcohols and ***S*-Por1_Zn_**. Hence, the influences of temperature and concentration on this ***S*-Por1_Zn_**-based supramolecular polymer in the presence of alcohols were examined. Since ***S*-Por1_Zn_** shares many structural features with ***S*-Por2_Cu_**, which showed the thermally bisignate assembly behavior in the presence of alcohols, we assume that the ***S*-Por1_Zn_** assembly is also heavily dependent on the temperature. This hypothesis was assessed by recording the absorption spectra in a temperature range from 90 to −5 °C while slowly cooling a solution of 10 μM ***S*-Por1_Zn_** with 4280 equiv of ethanol in MCH (Figure 3a). At 90 °C, this solution showed a distinct absorption maximum due to free ***S*-Por1_Zn_**. Upon cooling, a blue-shifted peak assignable to the H-type supramolecular aggregate appeared at 391 nm, which increased in intensity upon being cooled to 40 °C. Simultaneously, a peak due to free ***S*-Por1_Zn_** decreased, while a new absorption maximum appeared at 427 nm, which is attributable to the ethanol–***S*-Por1_Zn_** complex. Further cooling to −5 °C resulted in the increment of the absorption band due to the ethanol–***S*-Por1_Zn_** complex at the expense of the absorption band due to the H-aggregate. These observations indicate that ***S*-Por1_Zn_** can merely assemble into a helical supramolecular polymer in a specific temperature window and that the monomer is solvated outside this temperature window. At high temperatures, free ***S*-Por1_Zn_** was stabilized by the entropic penalty of the supramolecular polymerization that possibly outweighs the enthalpic energy gain, which is generally valid for supramolecular polymerizations. At low temperatures, the monomer was stabilized by competitive hydrogen bonding with ethanol by taking advantage of the potential enthalpic energy of monomeric ethanol in solution.^37^ This effect is identical to the polymer–monomer transition in the reported thermally bisignate polymerization.^25^ Thus, the presence of a temperature window with ***S*-Por1_Zn_** assemblies in between these different monomer states is in line with the thermally bisignate polymerization behavior.

Realizing similar sequestration effects of ***S*-Por1_Zn_** using ethanol and pyridine, we were intrigued to examine if the system with ethanol could also show the behavior of dilution-induced assembly. For this purpose, the effect of the concentration of ***S*-Por1_Zn_** on its assembly in MCH was investigated by means of absorption spectroscopy. A stock solution of 20 μM ***S*-Por1_Zn_** with 4280 equiv of ethanol was prepared and diluted with pure MCH to the desired concentration at a constant ***S*-Por1_Zn_**/ethanol ratio, and then, the samples were thermally equilibrated. The concentration-corrected absorption spectra showed that most of the monomers were solvated by ethanol at an ***S*-Por1_Zn_** concentration of 20 μM (Figure 3b). At an ***S*-Por1_Zn_** concentration of 10 μM, a minor peak due to the helical assembly of ***S*-Por1_Zn_** was observed, which became dominant when the concentrations of ***S*-Por1_Zn_** were lowered to 5 and 2 μM. These observations suggest that, similar to pyridine, ethanol can function as a concentration-dependent sequestrator for ***S*-Por1_Zn_**, facilitating its supramolecular polymerization upon dilution. However, ethanol binds to hydrogen-bonding motifs, which are prevalent in monomers for supramolecular polymerization, in contrast to pyridine−Zn coordination that is specific for porphyrin-type monomers with metal centers.

### Simulation of Assembly Landscapes by the Fitting Equilibrium Model to Experimental Data

**Figure 4:**
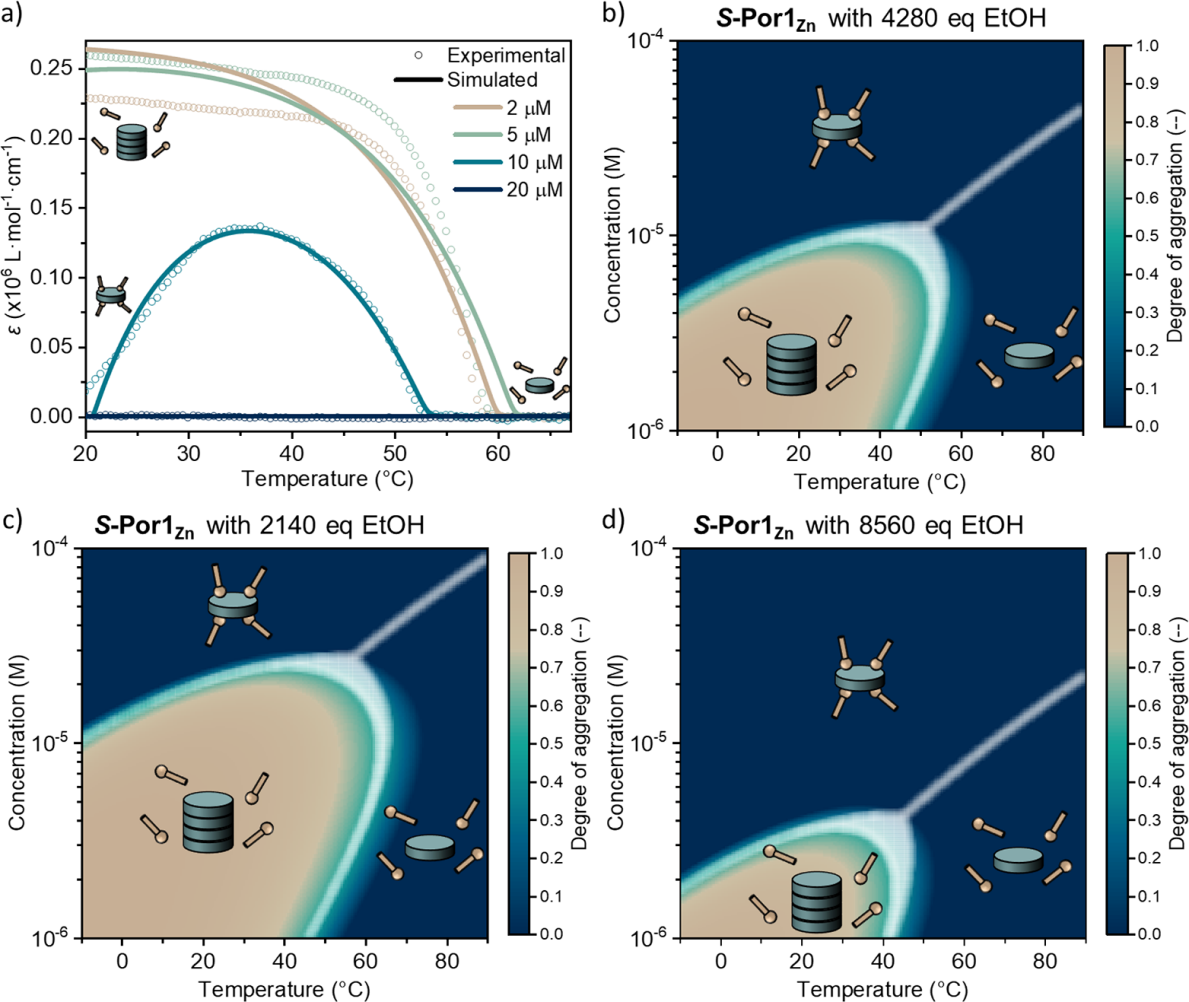
(a) Optimized fit (solid lines) of the theoretical model to the absorption cooling curves at [***S*-Por1_Zn_**] = 2, 5, 10, and 20 μM in MCH with 4280 equiv of EtOH (open circles) at 391 nm. Simulated assembly landscapes of ***S*-Por1_Zn_** with (b) 4280, (c) 2140, and (d) 8560 equiv of EtOH. The assembly landscapes are shown as a function of temperature (*x* axis) and Zn-porphyrin concentration (*y* axis). The colors indicate the degree of aggregation from 0 (dark blue) to 1 (light brown). The white regions indicate the transition areas between different states where no dominant species is present. The assembly landscapes unveil that the concentration and temperature effects on the supramolecular polymers of ***S*-Por1_Zn_** result from the same underlying interaction.

A series of the experiments described above demonstrated that the supramolecular polymer–solvent system responds differently to temperature at varied concentrations. As an attempt to understand the concentration and temperature effects on supramolecular assemblies, cooling curves of ***S*-Por1_Zn_** in MCH with 4280 equiv of ethanol were measured at various concentrations between 2 and 20 μM. Then, a thermodynamic mass-balance model was fitted to these cooling curves to extract the thermodynamic parameters of the ethanol–***S*-Por1_Zn_** interaction (Figure 4a). The obtained parameters were used to construct the assembly landscape of ***S*-Por1_Zn_** as a function of the concentration and temperature (Figure 4b). With the ethanol–***S*-Por1_Zn_** interaction occurring at higher concentrations, the landscape unveiled that the dilution-induced assembly can occur at any temperature below the elongation temperature (*T*_e_) of ***S*-Por1_Zn_**. In contrast, it appeared that the temperature effect discussed in Figure 3a only exists in a concentration range between 6 and 12 μM for this system. The assembly landscape clarifies that the concentration and temperature effects observed in Figure 3 are consequences of the same underlying interaction between ethanol and ***S*-Por1_Zn_**.

In view of the strong concentration dependency of the ethanol–***S*-Por1_Zn_** interaction, we were curious to investigate the effect of the amount of ethanol on the assembly landscape of ***S*-Por1_Zn_**. Hence, the assembly landscapes were constructed for the supramolecular polymer system with different amounts of ethanol with respect to the ***S*-Por1_Zn_** concentration, using the same mass-balance model. Figure 4c and Figure 4d display the assembly landscapes with 2140 and 8560 equiv of ethanol, respectively. The apparent shifts of the assembly landscape imply that the supramolecular polymer system can be finely tuned by simply adjusting the cosolvent composition.

To widen the scope of our research, we extended this experiment to other porphyrin-,^25^ triphenylamine-,^34,38^ benzene-,^39,40^ and triphenylene-based^21^ monomers that are decorated with amide moieties to provide hydrogen-bonding interactions and observed the occurrence of dilution-induced assembly in all these systems (Figure 1 and Figure S5). Therefore, the interaction of ethanol with the hydrogen-bonding motifs of monomers has been proven as a general approach to facilitate the dilution-induced assembly of hydrogen-bonded supramolecular polymers. This generality is evident from the assembly landscapes that were constructed for porphyrin ***S*-Por2_Zn_** and triphenylamine ***S*-TPA** in the presence of ethanol (Figures S4h and S7d). In general, this computational method is readily applicable, and the assembly landscapes provide immediate insights into the assembly properties of monomers under different conditions.

**Figure 5:**
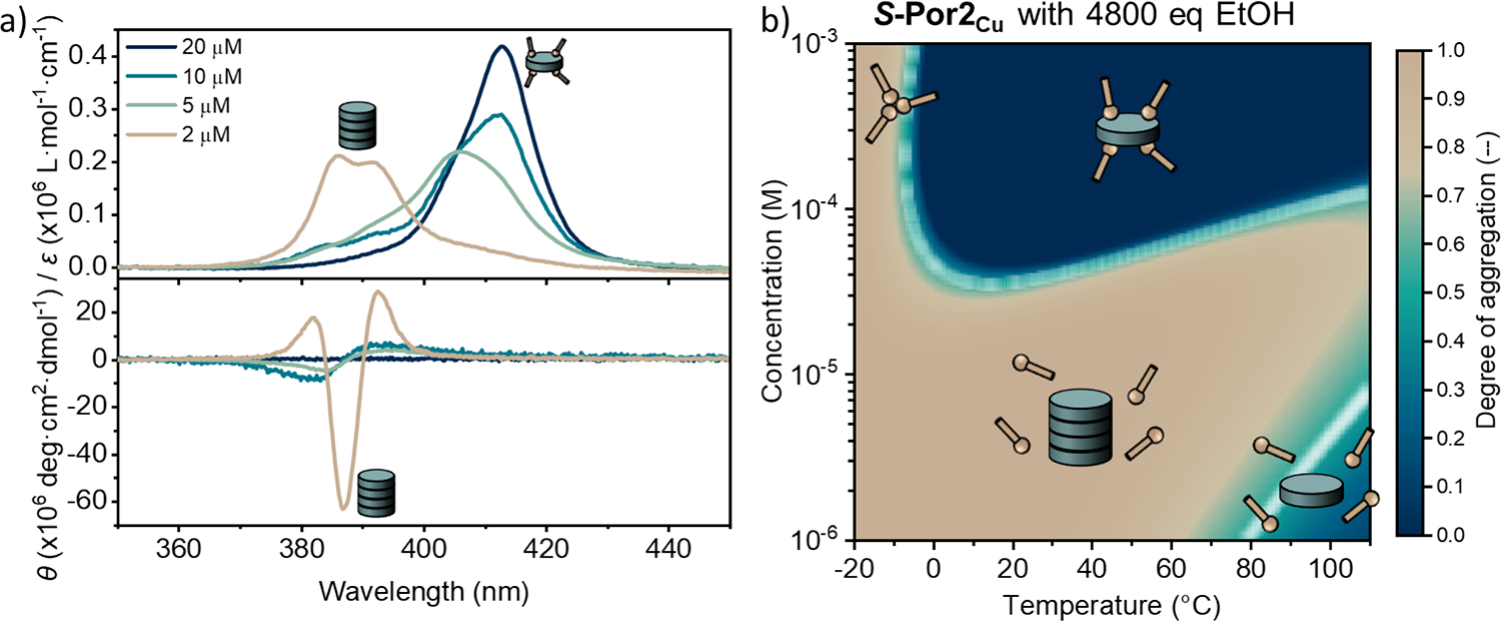
(a) Absorption (top) and ECD (bottom) spectra of the MCH solutions of ***S*-Por2_Cu_** with 34,250 equiv of EtOH at 20 °C; (b) simulated assembly landscape of ***S*-Por2_Cu_** with 4800 equiv of EtOH. The assembly landscape is shown as a function of temperature (*x* axis) and Cu-porphyrin concentration (*y* axis). The colors indicate the degree of aggregation from 0 (dark blue) to 1 (light brown). The white regions indicate the transition areas between different states where no dominant species is present. ***S*-Por2_Cu_** showed dilution-induced supramolecular polymerization. The assembly landscape unveiled that this is caused by the same alcohol–Cu-porphyrin interaction as the thermally bisignate polymerization.

The assembly landscape of ***S*-Por2_Cu_** attracted particular attention because this compound has shown both thermally bisignate and dilution-induced supramolecular polymerization (Figure 5a). Remarkably, the assembly landscape of ***S*-Por2_Cu_** with 4800 equiv of ethanol (Figure 5b) revealed that the thermally bisignate polymerization occurs exclusively in a narrow concentration window, which can be easily tuned by adjusting the alcohol excess that is present in the system. From these features, it becomes apparent that the depolymerization of ***S*-Por2_Cu_** at high concentrations and at intermediate temperatures relies on the same underlying interaction, which causes the dilution-induced and thermally bisignate assembly phenomena.

### Navigating Studies on the Supramolecular Polymer–Solvent System

**Figure 6:**
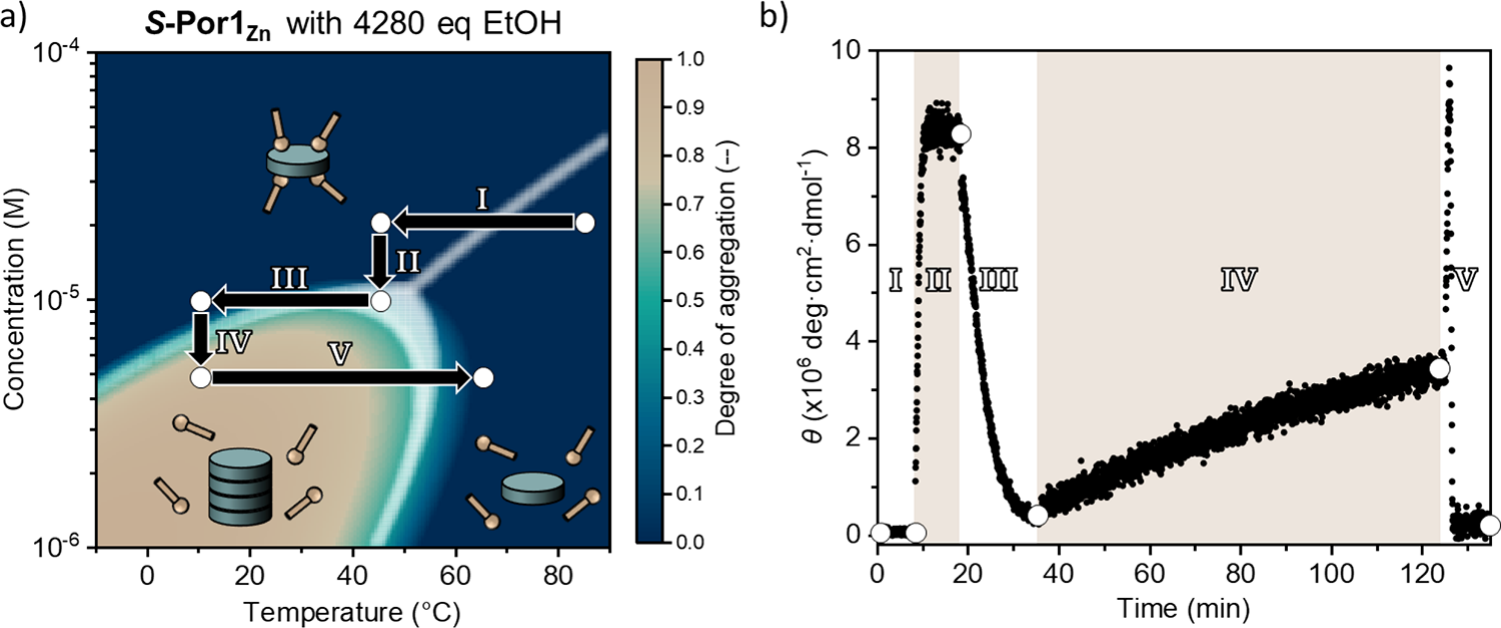
(a) Simulated assembly landscape of ***S*-Por1_Zn_** in MCH with 4280 equiv of EtOH. The black arrows and white circles indicate changes in conditions that were induced in the sample; (b) molar ellipticity of the supramolecular polymer of ***S*-Por1_Zn_** at 389 nm, monitored over time during the changes in conditions as indicated in panel (a). The observed transitions were expected based on the simulated assembly landscape, but the equilibration time for each step differed significantly.

Since the simulated assembly landscapes are based on computational models that assume thermodynamic equilibrium, the dynamicity and equilibration times of the supramolecular polymers were investigated experimentally. To navigate this specific assembly landscape, we quickly changed the sample concentration or temperature (Figure 6a, schematically illustrated with arrows in the simulated assembly landscape) and investigated the associated change in the CD spectrum of supramolecularly polymerized ***S*-Por1_Zn_** in MCH with ethanol (Figure 6b). To quickly change the concentration of ***S*-Por1_Zn_**, the sample was diluted with MCH and vigorously shaken immediately afterward to obtain a homogeneous sample. For making swift changes in temperature, we employed an uncontrolled temperature ramp at the maximum cooling/heating capacity of the CD instrument. Starting at 85 °C, an MCH solution of 20 μM ***S*-Por1_Zn_** with 4280 equiv of ethanol was cooled down to 45 °C (I), where no supramolecular polymers formed as expected. Diluting the sample to 10 μM (II) induces the assembly of ***S*-Por1_Zn_** to give supramolecular polymers that were equilibrated in less than 2 min. Subsequent cooling to 10 °C (III) resulted in the solvation of ***S*-Por1_Zn_** by ethanol, thereby disassembling its supramolecular polymers within 15 min. Dilution to 5 μM (IV) caused the second dilution-induced assembly, although the system was equilibrated significantly slower at 10 °C than in the previous dilution-induced assembly at 45 °C. After 90 min, when the sample was not yet fully equilibrated, we increased the temperature to 65 °C (V). During the heating ramp, we observed a rapid increase in the degree of aggregation within a minute, which was followed by an equally quick diminution of the CD signal when the temperature exceeded the *T*_e_ of ***S*-Por1_Zn_**. However, if the sample was left to fully equilibrate in stage IV for 130 min, then elevating the temperature to 65 °C in stage V led to the expected loss of the CD signal (Figure S8). The transitions observed in this experiment closely follow the simulated assembly landscape, although the equilibration times are not predictable with the thermodynamic model. Nonetheless, the assembly landscapes provide a valuable indication of the eventual system response to the applied changes in external conditions.

## Outlook and Conclusions

In this work, we have assessed possible effects of alcohols as hydrogen-bond scavengers on supramolecular polymers that possess multiple hydrogen bonds to form helical structures. The concentration- and temperature-dependent solvation events of monomers by the alcohol additives resulted in the disassembly of the supramolecular polymers. Computational techniques were employed for simulating the assembly landscape of the supramolecular polymer systems as a function of the concentration and temperature, which is helpful in rationalizing the observed assembly properties. Using these assembly landscapes, we quantitatively demonstrated that alcohols sequestrate the monomers predominantly at high concentrations of the system, most probable by interfering with the hydrogen-bonding interactions of the polymer. Therefore, it becomes evident that dilution-induced assembly^22^ and thermally bisignate polymerization^25^ are caused by the same underlying interaction mechanism, which has not been described before.

We have shown that the assembly landscapes can be tuned simply by adjusting the amount or type of alcohol additives. Furthermore, we showed that the assembly landscapes were successfully constructed for various hydrogen-bonding supramolecular polymers in combination with ethanol. Although this approach can be universal, a general notion of the interactions between the system components is required to derive the mass-balance model. We also hope that this report possibly inspires the related community to utilize the powerful combination of experiments and computations in supramolecular systems, ultimately helping in understanding and predicting the response of systems to multiple dimensions of the external conditions. Similar to the revolution in covalent polymer chemistry that followed the introduction of phase diagrams, assembly landscapes might enable us to build complex molecular systems with emerging functionalities as our understanding of supramolecular polymer systems increases.

## Supporting Information Available

Additional UV–vis and CD spectra, details of the thermodynamic model, additional results of the fitting procedures, and additional phase diagrams (PDF)
ZIP file containing the scripts (ZIP)
Scripts for fitting homopolymerization and multicomponent systems (PDF)
Scripts for simulation of assembly landscapes (PDF)
