# Supplementary material for: Simulating Assembly Landscapes for Comprehensive Understanding of Supramolecular Polymer–Solvent Systems
Source: J Am Chem Soc. 2023 Feb 9;145(7):4231–7. doi: 10.1021/jacs.2c12941 (PMC9951209; doi:10.1021/jacs.2c12941)
Supplement: Supplementary file 1 — ja2c12941_si_001.pdf [file ja2c12941_si_001.pdf]

Supporting Information for:

Simulating assembly landscapes for comprehensive understanding of supramolecular polymer–solvent systems

Stef A.H. Jansen,[1,2] Elisabeth Weyandt,[1,2] Tsubasa Aoki,[3] Takayoshi Akiyama,[3] Yoshimitsu Itoh,[3] Ghislaine Vantomme,[1,2] Takuzo Aida[3,4]* and E.W. Meijer[1,2,5]*

[1]Institute for Complex Molecular Systems, Eindhoven University of Technology, PO Box 513, 5600 MB, Eindhoven, The Netherlands. [2]Laboratory of Macromolecular and Organic Chemistry, Eindhoven University of Technology, P.O. Box 513, 5600 MB Eindhoven, The Netherlands. [3]Department of Chemistry and Biotechnology, School of Engineering, The University of Tokyo, 7-3-1 Hongo, Bunkyo-ku, Tokyo 113-8656, Japan. [4]RIKEN Center for Emergent Matter Science, 2-1 Hirosawa, Wako, Saitama 351-0198, Japan. [5]School of Chemistry and RNA Institute, UNSW, Sydney, Australia.

* To whom correspondence should be addressed: E-Mail: e.w.meijer@tue.nl

E-Mail: aida@macro.t.u-tokyo.ac.jp

Contents

S1. Experimental Methods **S3**

S2. Synthesis of *S*-Por1$_{Zn}$, *S*-Por2$_{Zn}$ and *S*-Por2$_{Cu}$ **S4**

S2.1 Synthesis of ***S*-Por1$_{Zn}$** S4

S2.2 Synthesis of ***S*-Por2$_{Zn}$** S4

S2.3 Synthesis of ***S*-Por2$_{Cu}$** S6

S3. Supplementary figures **S7**

Figure S1 ..... S7

Figure S2 ..... S8

Figure S3 ..... S9

Figure S4 ..... S10

Figure S5 ..... S12

Figure S6 ..... S13

Figure S7 ..... S13

Figure S8 ..... S14

S4. Details on the computational analyses **S14**

S4.1 Details on mass-balance models S14

S4.2 Schematic overview of included aggregation processes S17

Scheme S1 ..... S17

S4.3 Fitting procedure S17

S.4.4 Optimized fit parameters S18

Table S1 ..... S18

Table S2 ..... S19

Table S3 ..... S19

Table S4 ..... S19

S5. Workflow to simulate the assembly landscapes **S19**

References **S20**

S1. Experimental Methods

All starting materials were obtained from commercial suppliers and used without prior purification. The organic solvents used in spectroscopic experiments were of spectroscopic grade. ^{1}H NMR spectra were recorded on a JEOL model JNM-ECA 500II spectrometer operating at 500 MHz. All chemical shifts (δ) are expressed in ppm. ^{1}H NMR spectra are referenced relative to the signals arising from residual non-deuterated solvent, $CHCl_3$ (δ = 7.26 ppm). Matrix-assisted laser desorption/ionization time-of-flight (MALDI-TOF) mass spectrometry was performed in reflector mode on a Bruker model Autoflex™ speed spectrometer. Column chromatography was performed on a Biotage model Isolera™ Prime flash system, using a Biotage SNAP Cartridge KP-Sil or Ultra column (25–100 g; particle size 50 μm or 25 μm; irregular silica). Recycling preparative size exclusion chromatography (SEC) was performed on a JAI model LC-9130 recycling HPLC system equipped with a JAI model UV-600NEXT variable-wavelength UV/Vis detector. Circular Dichroism (CD) spectroscopy was performed on a JASCO J-815 CD spectrometer with a JASCO MPTC-490S thermostat. For full spectra measurements at variable temperature, the cooling ramp was halted during measurement. Gilson Microman pipettes were used to transfer solutions. Simulations were performed with the attached scripts on Matlab® R2020a.

Sample preparation for CD samples: Spectroscopic samples were prepared by weighing the desired compound into a screw-capped vial and adding the required amount of methylcyclohexane (MCH) to reach the desired concentration. The sample was then sonicated for approximately 1 minute and vortexed for 20 seconds to fully dissolve the compounds. In a separate vial, the desired alcohol was mixed with MCH and the solution was vortexed for 20 seconds. The samples were then prepared for measurement by pipetting the monomer solution, alcohol solution and MCH into quartz cuvettes with a pathlength of 1 or 10 millimeter. The samples were then vigorously shaken and equilibrated by heating the sample in the instrument. After subsequent controlled cooling with a rate of 1 K/min to the desired temperature, the spectra were collected.

Sample preparation for navigating assembly landscape measurements: For the experiment where the assembly landscapes are navigated within one solution, the samples were prepared in MCH as described. A quartz cuvette with a pathlength of 10 millimeter was loaded with 2.6 mL of MCH solution. The sample was heated to the initial measurement conditions for the experiment. For the cooling steps, the CD instrument was set to cool as quickly as possible to the desired temperature while collecting the kinetic trace. For the dilution steps, 1.3 mL of the solution was taken out and 1.3 mL of MCH was added to the cuvette. The sample was shaken vigorously and quickly placed in the CD instrument, where the kinetic trace was collected directly. After a plateau in each kinetic trace, the full spectra were acquired and then the next condition change was performed.

S2. Synthesis of *S*-Por1$_{Zn}$, *S*-Por2$_{Zn}$ and *S*-Por2$_{Cu}$

S2.1 Synthesis of ***S*-Por1$_{Zn}$**

A previously synthesized batch of ***S*-Por1$_{Zn}$** was used,[1] which was synthesized according to previous reports.[2]

S2.2 Synthesis of ***S*-Por2$_{Zn}$**

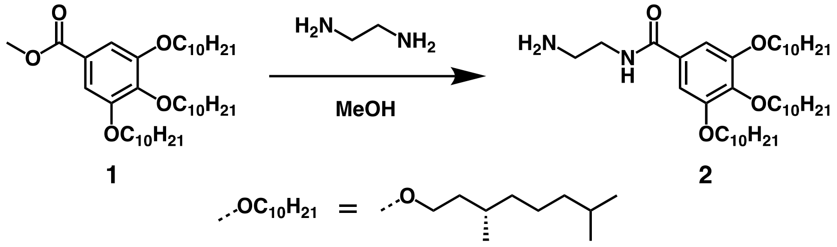

To **compound 1**[3,4] (211 mg, 0.348 mmol) and ethylene diamine (3.84 mL) was added MeOH (2.46 mL) in a microwave vial. The sealed vial was stirred at 120 °C for 3 h in a microwave reactor. The reaction mixture was concentrated under reduced pressure to give a crude wax. The crude was subjected to column chromatography on silica gel using DCM/MeOH (9/1 v/v) to give the product, **compound 2** (144 mg, 0.227 mmol) in 65 % yield. ^{1}H NMR (500 MHz, $CDCl_3$, 25 °C): δ (ppm) 7.00 (s, 2H), 6.65 (br, 1H), 4.08-3.98 (m, 6H), 3.50 (q, *J* = 5.7 Hz, 2H), 2.95 (t, *J* = 6.0 Hz, 2H), 1.89-1.50 (m, 12H), 1.33-1.11 (m, 18H), 0.94-0.86 (m, 27H).

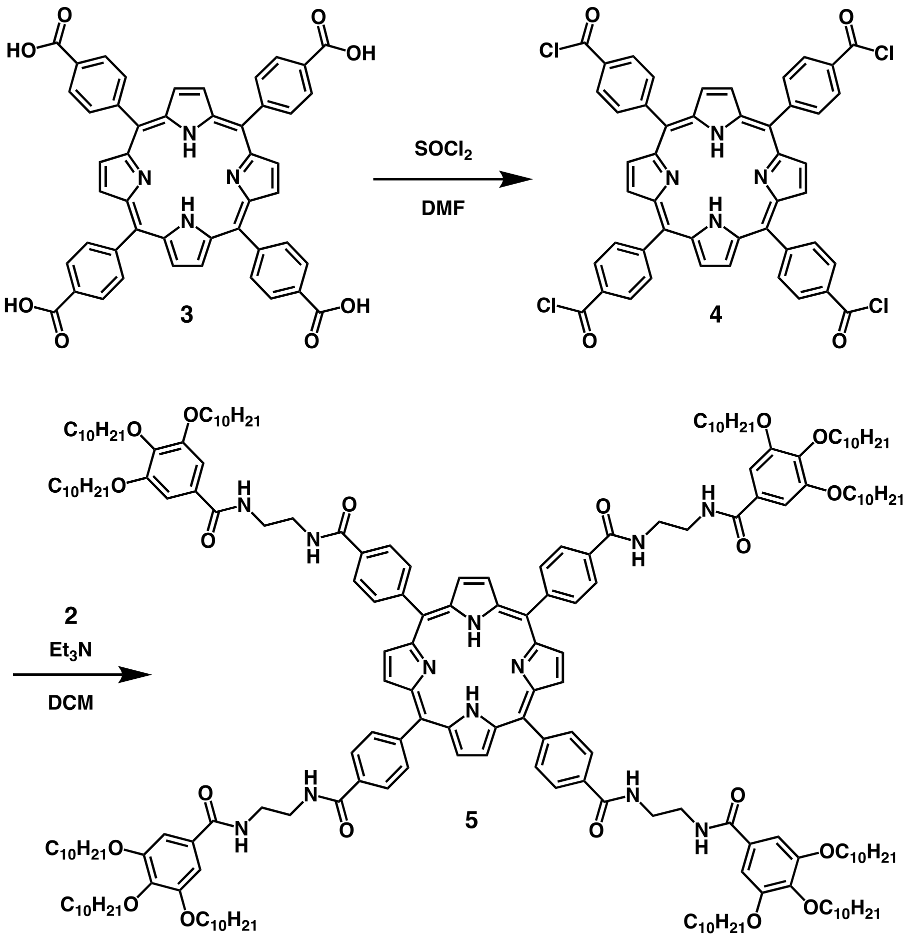

To tetrakis(4-carboxyphenyl)porphyrin (**compound 3**, 30.3 mg, 0.0383 mmol) was added thionyl chloride (2 mL) and one drop of dry DMF. The reaction mixture was refluxed for 5.5 h in the dark. After cooling to room temperature, the resultant solution was concentrated under reduced pressure to give a crude as greenish solids. The product, **compound 4** was used for the following steps without further purification.

To a dry DCM (1.8 mL) solution of **compound 4** at 0 °C was added **compound 2** in a dry DCM solution (136 mg, 0.214 mmol in 1.8 mL of DCM) and Et_3N (55 μL, 41 mg, 0.32 mmol). The reaction mixture was stirred at room temperature for 11 h. After dilution with DCM (10 mL), the resultant solution was poured into an aqueous solution of NH_4Cl and extracted with DCM. The combined organic phase was washed several times with 1 M NaOH solution and brine, dried over Na_2SO_4, and concentrated under reduced pressure. The residue was subjected to column chromatography on silica gel using DCM/MeOH (96/4 v/v) twice and followed by recycling size exclusion chromatography with $CHCl_3$ as an eluent to give the product, **compound 5** as purple solids (43.8 mg, 0.0135 mmol) in 35% yield. ^1H NMR spectrum was consistent with the previous report.[5] MALDI-TOF-MS (9-nitroanthracene as a matrix): *m/z*, 3248.29 ($[M]^+$ calcd. 3248.36)

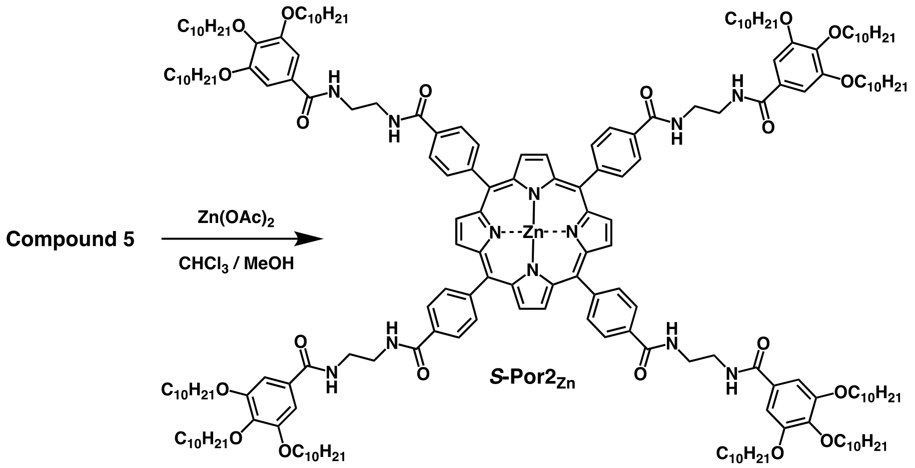

To **compound 5** (18.0 mg, 5.54 μmol) in a Schlenk tube was successively added $CHCl_3$ (3.7 mL), $Zn(OAc)_2$ (20.5 mg, 0.112 mmol), and MeOH (0.92 mL). The reaction mixture was stirred in the dark at 66 °C for 6 h in the sealed tube. After dilution with $CHCl_3$ (12 mL), the resultant mixture was filtered, followed by reprecipitation using DCM/MeOH to give the product, ***S*-Por2$_{Zn}$**, as purple solids (14.2 mg, 4.28 μmol) in 77% yield. ^{1}H NMR spectrum was consistent with the previous report.[5] MALDI-TOF-MS (9-nitroanthracene as a matrix): *m/z*, 3310.23 ($[M]^+$ calcd. 3310.27)

S2.3 Synthesis of ***S*-Por2$_{Cu}$**

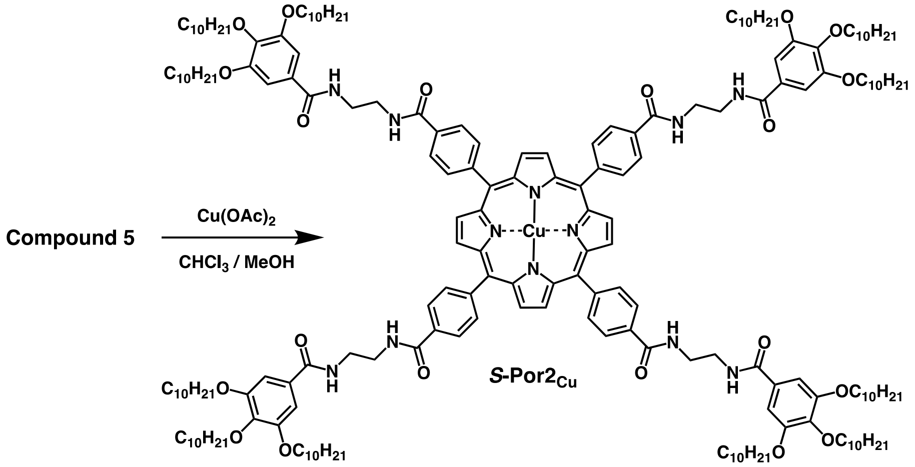

To **compound 5** (24.0 mg, 7.38 μmol) in a Schlenk tube was successively added $CHCl_3$ (4.96 mL), $Cu(OAc)_2$ (28.4 mg, 0.156 mmol), and MeOH (1.23 mL). The reaction mixture was stirred in the dark at 70 °C for 8 h in the sealed tube. After dilution with $CHCl_3$ (15 mL), the resultant mixture was filtered to give the product, ***S*-Por2$_{Cu}$** as red-purple solids in a quantitative yield.[5] MALDI-TOF-MS (9-nitroanthracene as a matrix): *m/z*, 3309.31 ($[M]^+$ calcd. 3309.27)

S3. Supplementary figures

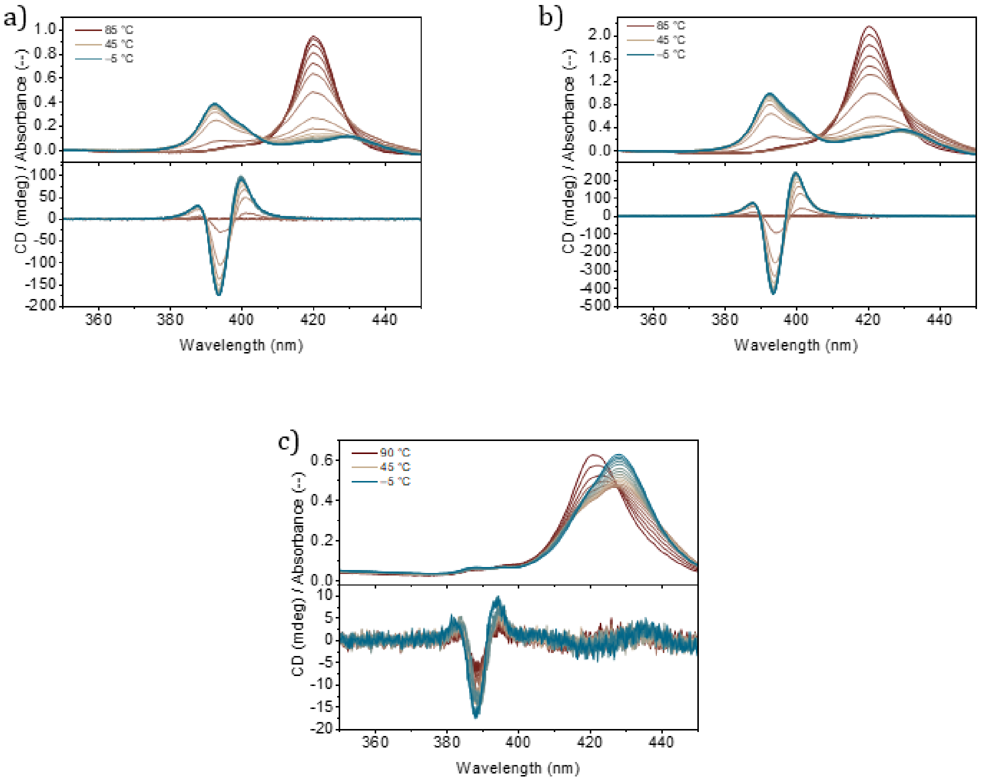

Figure S1: VT-absorption spectra (top) and VT-ECD spectra (bottom) of a) 2, b) 5 and c) 20 µM ***S*-Por1**$_{\mathbf{Zn}}$ with 4280 eq EtOH in MCH with a 1 K/min cooling rate. At 2 and 5 µM, a transition is observed from free ***S*-Por1**$_{\mathbf{Zn}}$ monomers at high temperatures to supramolecular polymers at low temperatures. At 20 µM, the transition from free ***S*-Por1**$_{\mathbf{Zn}}$ monomers at high temperatures to ethanol–monomer complexes at low temperatures is observed.

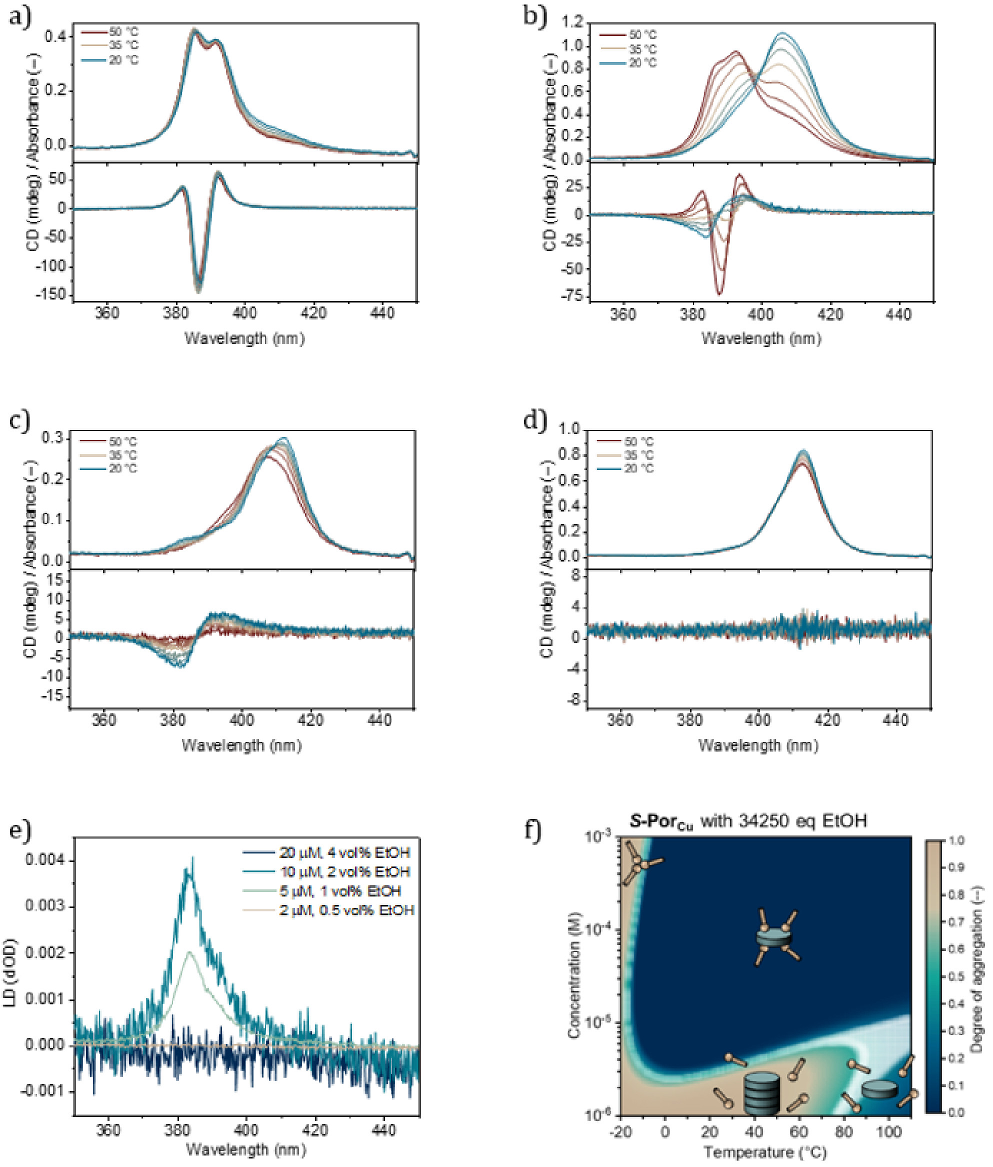

Figure S2: VT-absorption spectra (top) and VT-ECD spectra (bottom) of a) 2, b) 5, c) 10, and d) 20 µM ***S*-Por2**$_{Cu}$ with 34250 eq EtOH in MCH with 2 vol% $CHCl_3$ with a 0.5 K/min cooling rate. At 2 µM, ***S*-Por2**$_{Cu}$ is in the fully polymerized state over the whole temperature range. At 5 µM, a transition is observed from supramolecular polymers to ethanol–monomer complexes when the temperature is lowered. At 10 and 20 µM, ***S*-Por2**$_{Cu}$ is always in the depolymerized state. The CD signals of the 5 and 10 µM samples at low temperatures are attributed to an artefact of linear dichroism (LD), presumable due to the poor solubility of ***S*-Por2**$_{Cu}$ in MCH. e) LD of the samples of a-d at room temperature. f) Simulated assembly landscape of ***S*-Por2**$_{Cu}$ with 34250 eq EtOH in MCH with 2 vol% $CHCl_3$.

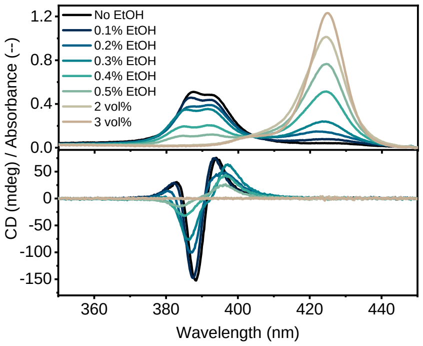

Figure S3: Absorption spectra (top) and ECD spectra (bottom) of 20 μM ***S*-Por2$_{Zn}$** with 0 to 3 vol% EtOH at 20 °C in MCH with a 0.5 K/min cooling rate equilibration. The spectral changes in the absorbance and the decrease of CD signal upon addition of more ethanol indicates that the supramolecular polymers of ***S*-Por2$_{Zn}$** are depolymerized by solvation of the free monomer.

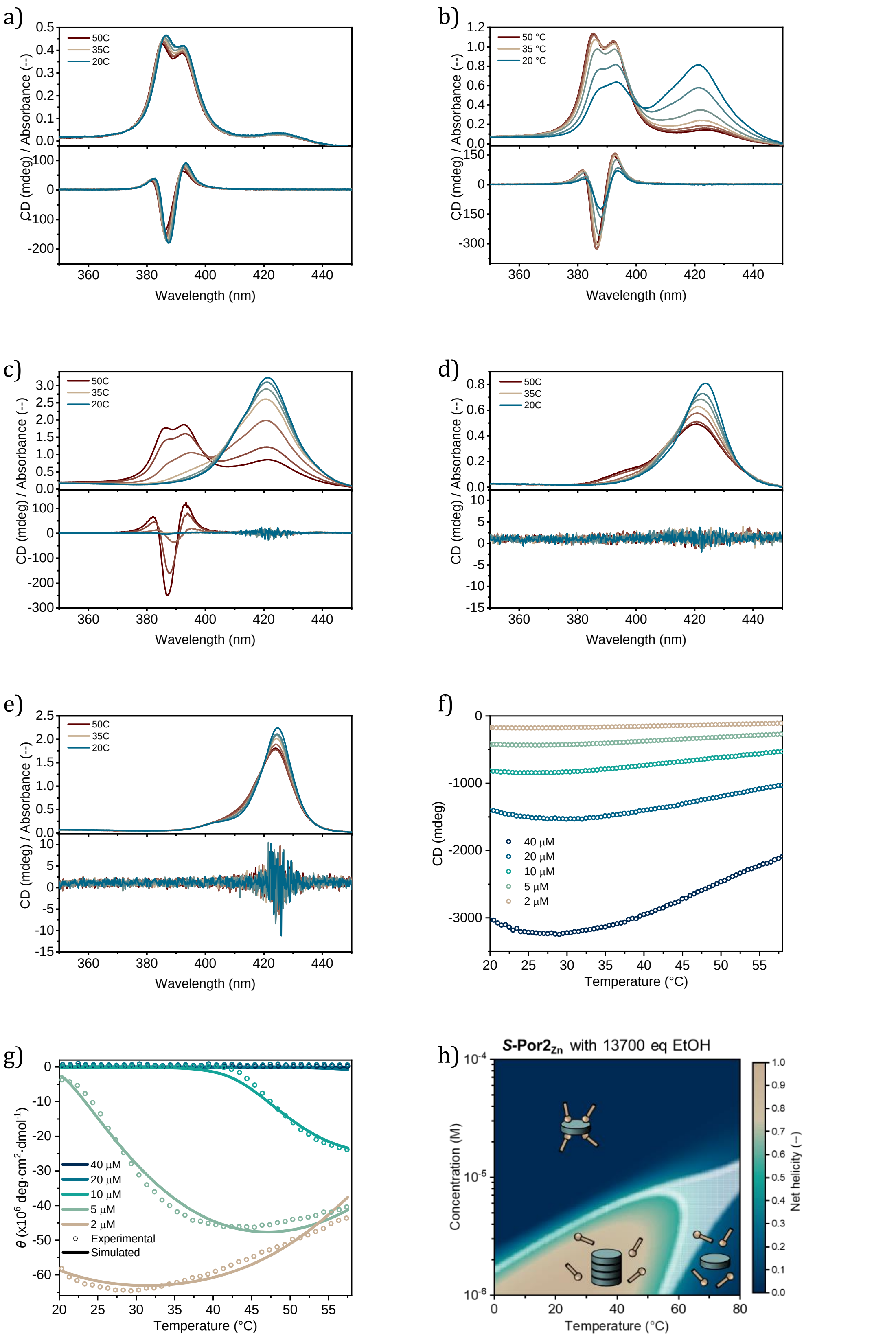

Figure S4: VT-absorption spectra (top) and VT-ECD spectra (bottom) of a) 2, b) 5, c) 10, d) 20 and e) 40 μM ***S*-Por2**$_{\mathbf{Zn}}$ with 13700 eq EtOH in MCH with 2 vol% $CHCl_3$ with a 0.5 K/min cooling rate. f) CD cooling curves (open circles) of [***S*-Por2**$_{\mathbf{Zn}}$] = 40, 20, 10, 5, and 2 μM in MCH

with 2 vol% $CHCl_3$ at 387 nm, g) optimized fit (solid lines) of the theoretical model to CD cooling curves (open circles) of [***S*-Por2$_{Zn}$**] = 40, 20, 10, 5, and 2 μM with 13700 eq EtOH in MCH with 2 vol% $CHCl_3$ at 387 nm and h) simulated assembly landscape of ***S*-Por2$_{Zn}$** with 13700 eq EtOH in MCH. At 2 μM, ***S*-Por2$_{Zn}$** forms supramolecular polymers at all monitored temperatures. At 5, 10 and 20 μM, a transition is observed from supramolecular polymers at high temperatures to solvated monomers at low temperatures. At 40 μM, ***S*-Por2$_{Zn}$** monomer is solvated in the entire temperature window that is monitored.

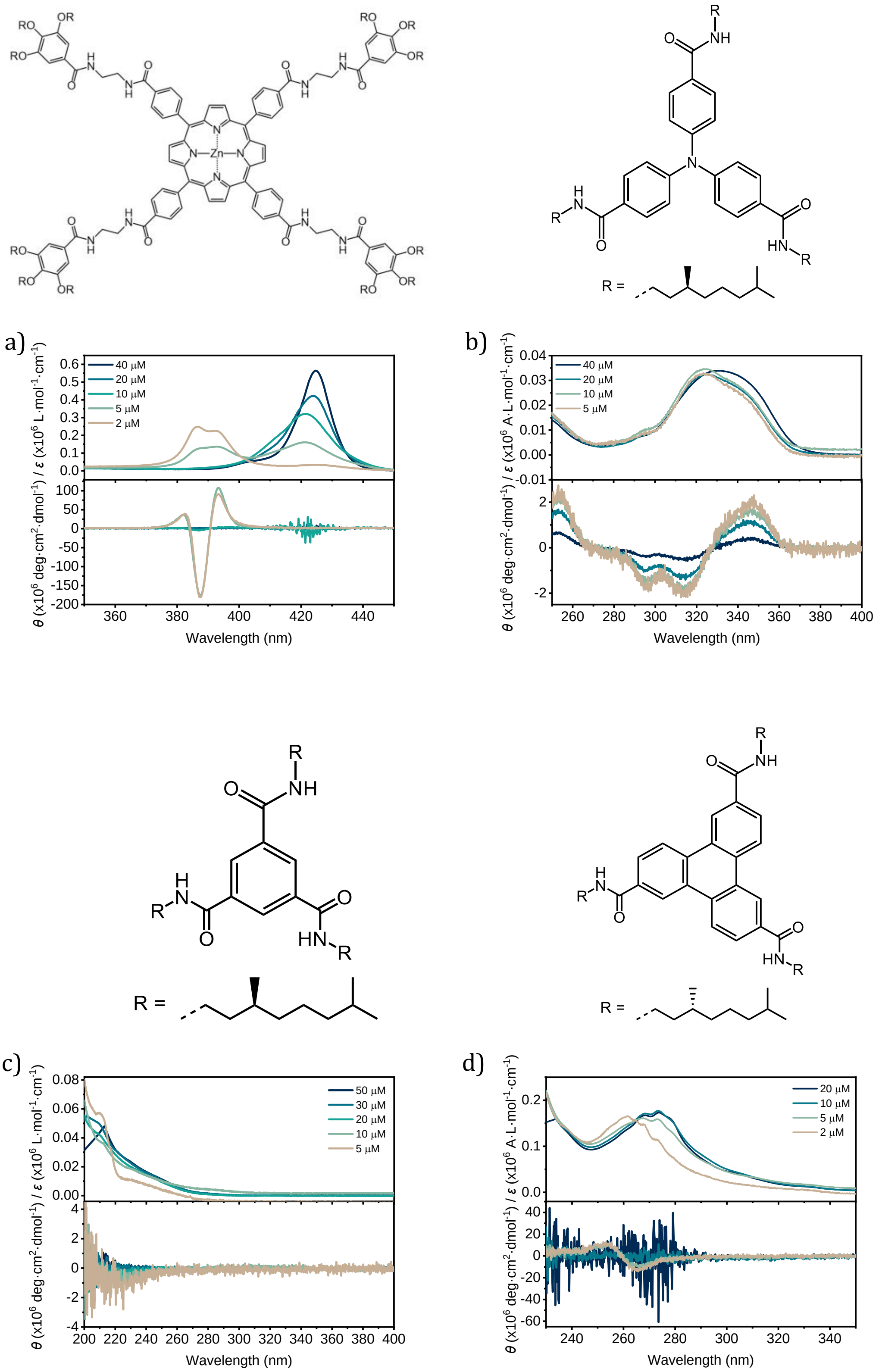

Figure S5: Molecular structures (above graph), absorption spectra (top) and ECD spectra (bottom) of a) ***S*-Por2$_{Zn}$**[5] with 13700 eq EtOH in MCH with 2 vol% $CHCl_3$, b) ***S*-TPA**[6] with 2140 eq EtOH in MCH, c) ***S*-BTA**[7] with 685 eq EtOH in MCH and d) ***R*-TTA**[8] with 17120 eq EtOH in decalin

at 20 °C. All compounds show a higher molar ellipticity at lower concentrations, indicating the ability to induce supramolecular polymerization by diluting the sample.

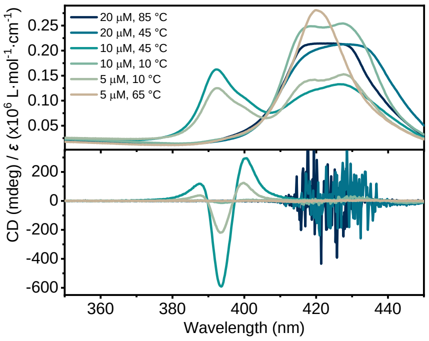

Figure S6: Absorption spectra (top) and ECD spectra (bottom) of ***S*-Por1$_{Zn}$** with 4280 eq EtOH in MCH during navigating experiments. The full spectra display the transitions as indicated in the assembly landscape in Figure 6a. At 20 µM the detector was oversaturated since the concentration was too high for the 10 mm cuvettes, but the spectra seem to indicate a shift towards longer wavelengths when changing the temperature from 85 to 45 °C. This is in line with spectral changes previously observed when going from free ***S*-Por1$_{Zn}$** monomer to monomer that is solvated by the alcohol additive (Figure S1c).

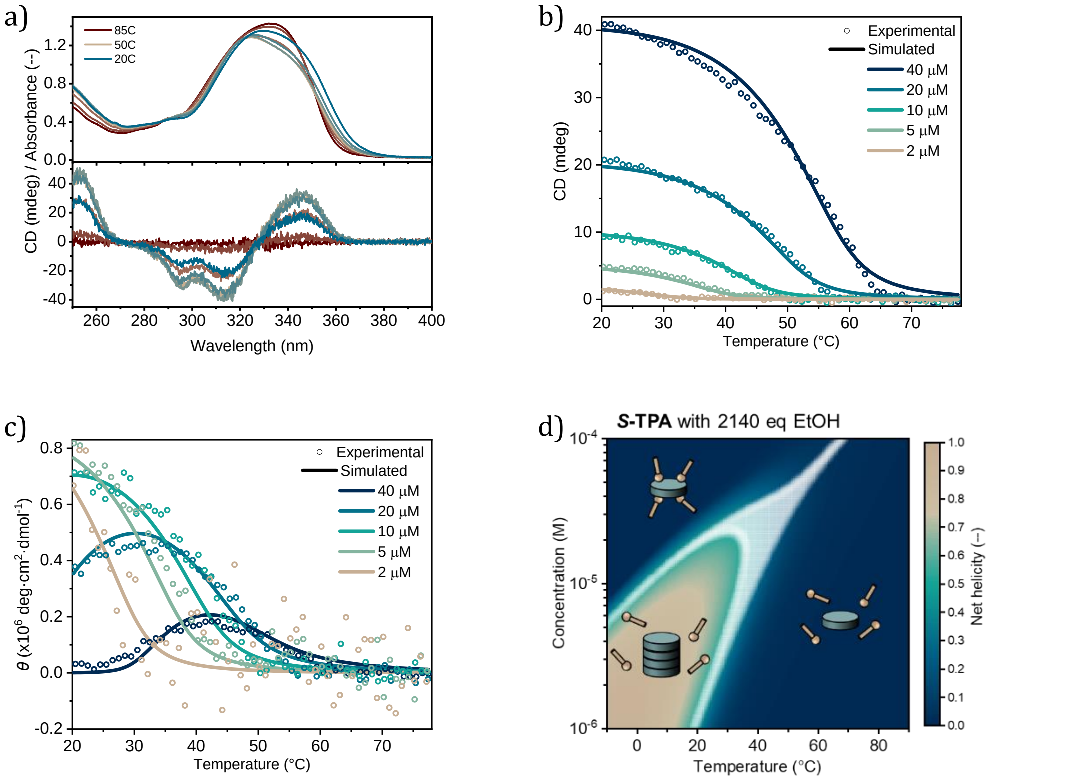

Figure S7: a) VT-absorption spectra (top) and VT-ECD spectra (bottom) of 40 µM ***S*-TPA** with 2140 eq EtOH with a 1 K/min cooling rate. Optimized fit (solid lines) of theoretical model to

CD cooling curves (open circles) of b) [***S*-TPA**] = 40, 20, 10, 5, and 2 μM in MCH at 346 nm, c) [***S*-TPA**] = 40, 20, 10, 5, and 2 μM with 2140 eq EtOH in MCH at 346 nm and d) simulated assembly landscape of ***S*-TPA** with 2140 eq EtOH in MCH. Similar to Figure 4a, the spectra show a maximum CD signal at intermediate temperatures that decreases upon cooling. The assembly landscape reveals that this is caused by the solvation of the ***S*-TPA** monomer by the alcohol additive at lower temperatures.

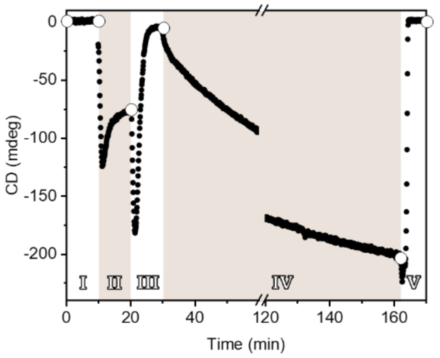

Figure S8: Kinetic traces of CD signal of **S-Por1$_{Zn}$** supramolecular polymers at 392 nm, monitored during changes in conditions as indicated in Figure 6a. This experiment was a reproduction of the experiment performed for Figure 6b, but this time followed at the minimum in the CD spectrum. The transition in stage II shows overshooting of the CD signal before equilibration, which is due to dilution with room temperature MCH, thus affecting the final solution temperature significantly. In stage IV, after a longer equilibration phase, the full CD signal is obtained.

S4. Details on the computational analyses

S4.1 Details on mass-balance models

The cooperative pathway in supramolecular polymerizations is modelled using thermodynamic mass balance expressions.[9] In the model, the polymers (H) are assumed to grow through monomer (M) addition and dissociation at the chain ends. The reactions that describe the cooperative pathway, for which a nucleus size of 2 is assumed, are:

$$\begin{aligned}
&\mathrm{M}+\mathrm{M} \underset{}{\overset{K_n}{\rightleftharpoons}} \mathrm{H}_2 \\
&\mathrm{H}_2+\mathrm{M} \underset{}{\overset{K_e}{\rightleftharpoons}} \mathrm{H}_3 \\
&\mathrm{H}_3+\mathrm{M} \underset{}{\overset{K_e}{\rightleftharpoons}} \mathrm{H}_4 \\
&\quad\vdots \\
&\mathrm{H}_j+\mathrm{M} \underset{}{\overset{K_e}{\rightleftharpoons}} \mathrm{H}_{j+1} \\
&\quad\vdots
\end{aligned} \tag{S1}$$

with K_n the nucleation constant and K_e the elongation constant of the nucleated pathway. Assuming the activity of the chemical species is equal to their concentrations, the concentration of monomers in i-mer in the cooperative H-aggregates in thermodynamic equilibrium can then be expressed as a function of the free monomer concentration with:

$$[\mathrm{H}_i] = i \cdot \sigma \cdot K_\mathrm{e}^{i-1} \cdot [\mathrm{M}]^i \text{ for } i \geq 2 \tag{S2}$$

where [M] is the equilibrium monomer concentration and σ is the cooperativity parameter, which is $\sigma = K_n/K_e$,.

The total concentration of M in the system is the sum of monomers and nucleated aggregates:

$$\begin{aligned}[\mathrm{M}]_\mathrm{tot} &= [\mathrm{H}]_\mathrm{tot} + [\mathrm{M}] \\ &= \left(\sum_{i=2}^{\infty}[\mathrm{H}_i]\right) + [\mathrm{M}] \\ &= \left(\sum_{i=1}^{\infty} i \cdot \sigma \cdot K_\mathrm{e}^{i-1}[\mathrm{M}]^i\right) - \sigma \cdot [\mathrm{M}] + [\mathrm{M}]\end{aligned} \tag{S3}$$

The subtraction of the monomer concentration compensates for double counting of the monomers in both sums.

With standard expressions for converging series, the summation in equation (S3) can be solved and the mass-balance equation for the system can be obtained:

$$[\mathrm{M}]_\mathrm{tot} = (1-\sigma)\cdot[\mathrm{M}] + \frac{\sigma \cdot [\mathrm{M}]}{(1-K_\mathrm{e}\cdot[\mathrm{M}])^2} \tag{S4}$$

This equation is solved in Matlab®, using a custom written binary search algorithm, to obtain the free monomer concentration. The free monomer concentration is then used to calculate the concentration of nucleated aggregates.

The solvent dependence of K_e to a cosolvent is introduced via:[10]

$$K_\mathrm{e} = \exp\left(\frac{-\Delta G_\mathrm{coop}}{R \cdot T}\right) = \exp\left(\frac{-(\Delta G_\mathrm{coop}^{\circ} + m_\mathrm{coop} \cdot f_\mathrm{cosolv})}{R \cdot T}\right) \tag{S5}$$

with R the gas constant, T the temperature, $\Delta G_{coop}°$ the Gibbs free energy of elongation of the cooperative polymerization, ΔG_{coop} the cosolvent-corrected Gibbs free energy of elongation of the cooperative polymerization and m_{coop} the solvent dependency parameter of the elongation process to the cosolvent that is present in solvent fraction f_{cosolv}.

The binding constant K_e is rendered temperature-dependent through the van 't Hoff expression:

$$K_{\mathrm{e}} = \exp\left(\frac{-\Delta G_{\mathrm{coop}}}{R \cdot T}\right) = \exp\left(\frac{-\Delta H_{\mathrm{coop}} - m_{\mathrm{coop}} \cdot f_{\mathrm{cosolv}}}{R \cdot T} + \frac{\Delta S_{\mathrm{coop}}}{R}\right) \tag{S6}$$

with ΔH_{coop} and ΔS_{coop} the enthalpy and entropy of elongation, respectively.

In the algorithm used to fit the melting curves, the nucleation enthalpy, ΔH_{n}, is introduced via:

$$\Delta H_{\mathrm{n}} = \Delta H_{\mathrm{coop}} + NP \tag{S7}$$

with ΔH_{coop} the enthalpy of elongation and NP a nucleation penalty. The nucleation penalty is related to the cooperativity parameter σ via:

$$\sigma = e^{\frac{-NP}{R \cdot T}} \tag{S8}$$

For the cooperative pathway, the nucleation energy, ΔG_{n}, is introduced via:

$$\Delta G_{\mathrm{n}} = \Delta G_{\mathrm{coop}} - R \cdot T \ln(\sigma) \tag{S9}$$

which relates to the nucleation constant via:

$$K_{\mathrm{n}} = \exp\left(\frac{-\Delta G_{\mathrm{n}}}{R \cdot T}\right) \tag{S10}$$

For isodesmic aggregation pathways, the equations above apply with σ=1, NP=0 and thus K_{n}=K_{e}. The clustering of ethanol in MCH was previously fitted as an isodesmic assembly, of which we will use the thermodynamic parameters ΔH_{clus} and ΔS_{clus}.

The interaction between the monomer M and additive S is considered as a sequential addition of S to M up to a 1:k complex MS_k:[11]

$$\begin{aligned}
\mathrm{M} + \mathrm{S} &\xrightleftharpoons{K_{\mathrm{MS}}} \mathrm{MS} \\
\mathrm{MS} + \mathrm{S} &\xrightleftharpoons{K_{\mathrm{MS}}} \mathrm{MS}_2 \\
\mathrm{MS}_2 + \mathrm{S} &\xrightleftharpoons{K_{\mathrm{MS}}} \mathrm{MS}_3 \\
&\vdots \\
\mathrm{MS}_{k-1} + \mathrm{S} &\xrightleftharpoons{K_{\mathrm{MS}}} \mathrm{MS}_k
\end{aligned} \tag{S11}$$

with K_{MS} the binding constant of additive to monomer. It is assumed that k equals the number of amide functional groups in the monomer.

The concentration of monomers in monomer–additive complexes in thermodynamic equilibrium $[\mathrm{MS}_k]_{tot}$ can then be expressed as a function of the free monomer concentration with:

$$[\mathrm{MS}_k]_{tot} = \sum_{i=1}^{k} K^{i}_{\mathrm{MS}} \cdot [\mathrm{M}] \cdot [\mathrm{S}]^{i} = [\mathrm{M}] \cdot \frac{K_{\mathrm{MS}} \cdot [\mathrm{S}] - (K_{\mathrm{MS}} \cdot [\mathrm{S}])^{k+1}}{1 - K_{\mathrm{MS}} \cdot [\mathrm{S}]} \tag{S12}$$

Adding this term to the mass-balance equation (S4) yields the mass-balance equation for monomer–additive systems:

$$[\mathrm{M}]_{\mathrm{tot}} = (1-\sigma)\cdot[\mathrm{M}] + \frac{\sigma\cdot[\mathrm{M}]}{(1-K_{\mathrm{e}}\cdot[\mathrm{M}])^2} + [\mathrm{M}]\cdot\frac{K_{\mathrm{MS}}\cdot[\mathrm{S}]-(K_{\mathrm{MS}}\cdot[\mathrm{S}])^{k+1}}{1-K_{\mathrm{MS}}\cdot[\mathrm{S}]} \qquad \text{(S13)}$$

The binding constant K_{MS} is rendered temperature-dependent through the van ‘t Hoff expression:

$$K_{\mathrm{MS}} = \exp\left(\frac{-\Delta G_{\mathrm{MS}}}{R\cdot T}\right) = \exp\left(\frac{-\Delta H_{\mathrm{MS}}}{R\cdot T}+\frac{\Delta S_{\mathrm{MS}}}{R}\right) \qquad \text{(S14)}$$

with ΔH_{MS} and ΔS_{MS} the enthalpy and entropy of the monomer–additive interaction, respectively.

S4.2 Schematic overview of included aggregation processes

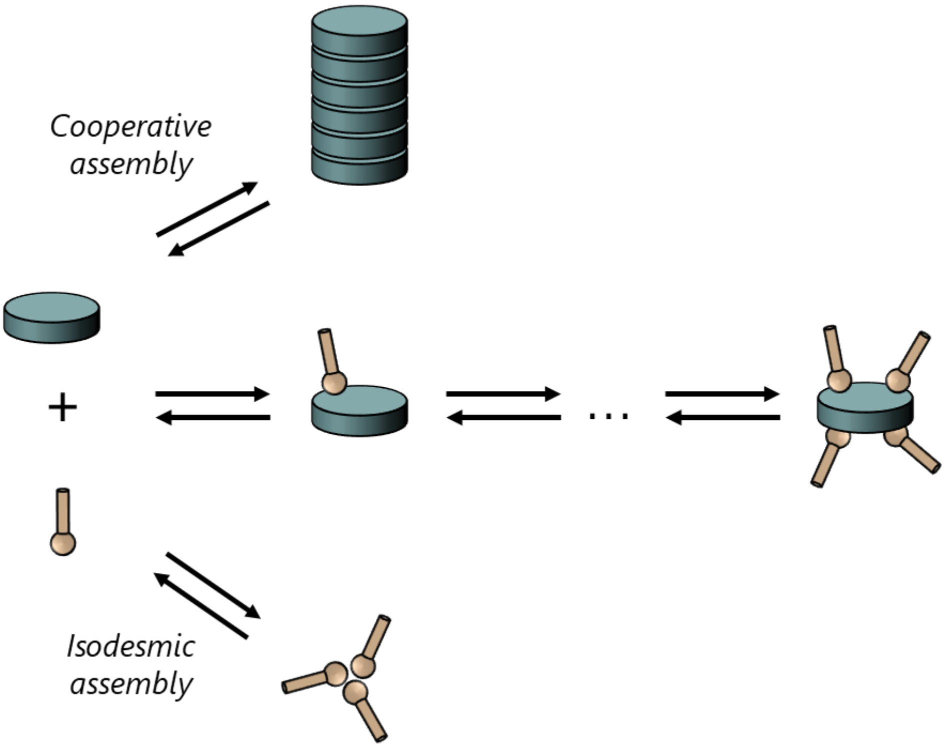

Scheme S1: Schematic representation of aggregation pathways included in the model for ***S*-Por1$_{\mathrm{Zn}}$** (green disc) with ethanol (brown stick) additive.

S4.3 Fitting procedure

The above-described model is fitted to the CD signal at the wavelength of the maximum, the absorbance of the polymer maximum A^{pol} and the absorbance of the monomer maximum A^{mon}. To predict the spectroscopic response, the concentration of every aggregate type is multiplied by the molar absorbance or molar ellipticity for the specific aggregate types:

$$\theta = \theta_{\mathrm{M}} \cdot [\mathrm{M}] \cdot L + \theta_{\mathrm{H}} \cdot [\mathrm{H}]_{\mathrm{tot}} \cdot L$$
$$A^{\mathrm{pol}} = \varepsilon_{\mathrm{M}}^{\mathrm{pol}} \cdot [\mathrm{M}] \cdot L + \varepsilon_{\mathrm{H}}^{\mathrm{pol}} \cdot [\mathrm{H}]_{\mathrm{tot}} \cdot L \qquad \text{(S1)}$$
$$A^{\mathrm{mon}} = \varepsilon_{\mathrm{M}}^{\mathrm{mon}} \cdot [\mathrm{M}] \cdot L + \varepsilon_{\mathrm{H}}^{\mathrm{mon}} \cdot [\mathrm{H}]_{\mathrm{tot}} \cdot L$$

where θ is the ellipticity in mdeg, θ_i is the molar ellipticity of species i in mdeg·M·cm^{-1}, A^{λ} is the absorbance at wavelength λ, $\varepsilon_x{}^{\lambda}$ is the molar absorbance of species x at the absorption wavelength of species λ and L is the optical path length of the cuvette. The molar ellipticity of the monomers θ_{M} is fixed at 0. θ_{H}, $\varepsilon_{\mathrm{H}}{}^{\mathrm{pol}}$, $\varepsilon_{\mathrm{H}}{}^{\mathrm{mon}}$ $\varepsilon_{\mathrm{M}}{}^{\mathrm{pol}}$, $\varepsilon_{\mathrm{M}}{}^{\mathrm{mon}}$ are estimated from the concentration-corrected experimental spectra. The fit parameters for the homopolymerizations are ΔH_{coop}, ΔS_{coop}, NP. The fit parameters for monomer–additive samples are m_{coop}, $\Delta H_{\mathrm{mon-add}}$ and $\Delta S_{\mathrm{mon-add}}$.

The predicted curves were fitted to the experimental data using least-squares minimization. The experimentally obtained data was compared with the predicted curves to obtain a cost vector:

$$Cost = \left[\left(\theta_{\mathrm{model}}(c,T) - \theta_{\mathrm{experiment}}(c,T) \right), \left(A_{\mathrm{model}}^{392\ \mathrm{nm}}(c,T) - A_{\mathrm{experiment}}^{392\ \mathrm{nm}}(c,T) \right), \left(A_{\mathrm{model}}^{425\ \mathrm{nm}}(c,T) - A_{\mathrm{experiment}}^{425\ \mathrm{nm}}(c,T) \right) \right] \qquad \text{(S2)}$$

The minimization of the cost vector is performed using the Matlab® lsqnonlin function with the trust-region-reflective algorithm to obtain optimal values for the thermodynamic parameters of the aggregation processes and spectroscopic properties of the aggregates.

To ensure that the solution is at the global minimum, the fits were performed with a minimum of at least 500 initial parameter sets. The initial parameter sets were defined using a Latin Hypercube Sampling method, implemented with the Matlab® function lhsdesign. To ensure reasonable values of the set of starting parameters in the fitting of the melting curves, Gibbs free energies of aggregation were sampled between –20 and –50 kJ/mol and ΔS_{coop} was sampled between –50 and –150 J/mol/K. From these sampled values the Gibbs free energies and entropies, reasonable random enthalpies were obtained. The final parameter set that resulted in the lowest norm of the residual cost vector was selected as the best fit.

S.4.4 Optimized fit parameters

Table S1: Thermodynamic parameters for ***S*-Por1$_{\mathbf{Zn}}$** with EtOH in MCH. The parameters in bold were obtained from the fitting procedure. The parameters not in bold were set as boundaries in the fitting procedure to keep the parameters in a realistic parameter range.

ΔH_{coop} (kJ/mol)	**ΔS_{coop} (J/mol/K)**	NP (kJ/mol)	m_{coop} (kJ/mol)	**ΔH_{MS} (kJ/mol)**	**ΔS_{MS} (J/mol/K)**	**ΔH_{clus} (kJ/mol)**	**ΔS_{clus} (J/mol/K)**
–70	**–97.6**	20	2000	**–35.6**	**–89.6**	**–9.7**	**–248.0**

Table S2: Thermodynamic parameters for ***S*-Por2$_{Cu}$** with EtOH in MCH/$CHCl_3$ 98/2 (v/v). The parameters were previously determined.[11] A good fit was obtained with an isodesmic assembly model without the solvent dependency parameter *m*.

ΔH_{coop} (kJ/mol)	ΔS_{coop} (J/mol/K)	*NP* (kJ/mol)	m_{coop} (kJ/mol)	ΔH_{MS} (kJ/mol)	ΔS_{MS} (J/mol/K)	ΔH_{clus} (kJ/mol)	ΔS_{clus} (J/mol/K)
−73.2	−79.8	0	0	−27.9	−50.8	−37.9	−103

Table S3: Thermodynamic parameters for ***S*-Por2$_{Zn}$** with EtOH in MCH/$CHCl_3$ 98/2 (v/v). The parameters in bold were obtained from the fitting procedure. A good fit was obtained without the solvent dependency parameter *m*. For the enthalpy and entropy of ethanol clustering (ΔH_{clus} and ΔS_{clus}, respectively), the values obtained from the fitting procedure of ***S*-Por2$_{Cu}$** (Table S2) were used that were obtained for the same solvent.

**ΔH_{coop} (kJ/mol)**	**ΔS_{coop} (J/mol/K)**	***NP* (kJ/mol)**	m_{coop} (kJ/mol)	**ΔH_{MS} (kJ/mol)**	**ΔS_{MS} (J/mol/K)**	ΔH_{clus} (kJ/mol)	ΔS_{clus} (J/mol/K)
**−107.1**	**−209.2**	**3.5**	0	**−43.7**	**−110.1**	−37.9	−103

Table S4: Thermodynamic parameters for ***S*-TPA** with EtOH in MCH. The parameters in bold were obtained from the fitting procedure. A good fit was obtained without the solvent dependency parameter *m*. For the enthalpy and entropy of ethanol clustering (ΔH_{clus} and ΔS_{clus}, respectively), the values obtained from the fitting procedure of ***S*-Por1$_{Zn}$** (Table S1) were used that were obtained for the same solvent.

**ΔH_{coop} (kJ/mol)**	**ΔS_{coop} (J/mol/K)**	***NP* (kJ/mol)**	m_{coop} (kJ/mol)	**ΔH_{MS} (kJ/mol)**	**ΔS_{MS} (J/mol/K)**	ΔH_{clus} (kJ/mol)	ΔS_{clus} (J/mol/K)
**−91.8**	**−194.2**	**8.7**	0	**−51.6**	**−143.2**	−9.7	−248.0

S5. Workflow to simulate the assembly landscapes

1. Measure VT-absorption and VT-ECD cooling curves of the monomer without additives at different concentrations.
2. Fit the thermodynamic model for cooperative polymerizations to the cooling curves of step 1 to obtain the thermodynamic parameters of the supramolecular polymerization. The fitting procedure is described in the previous section.

3. Measure full VT-absorption and VT-ECD spectra of the monomer with different amounts of the additive in the temperature range of interest. The sample with lowest additive content should be in a fully polymerized state at room temperature and the sample with the highest additive content should be just in the fully depolymerized state at room temperature.
4. Measure VT-absorption and VT-ECD cooling curves of the sample with the highest additive concentration of step 3 and dilute the sample to a few different concentrations within the concentration window of interest.
5. Fit the thermodynamic model for cooperative polymerization in the presence of an additive to the cooling curves of step 4 to obtain the thermodynamic parameters of the additive–monomer interaction. For this step, the thermodynamic parameters of the supramolecular polymerization from step 2 should be put into the model. The fitting procedure is described in the previous section.
6. Use the parameters obtained from the fitting procedures of step 2 and 5 as input in the assembly landscape script. Specify the concentration- and temperature-windows of interest.

References

1. Weyandt, E.; Leanza, L.; Capelli, R.; Pavan, G. M.; Vantomme, G.; Meijer, E. W. Controlling the length of porphyrin supramolecular polymers via coupled equilibria and dilution-induced supramolecular polymerization. *Nat. Commun.* **2022**, *13*, 1–9.
2. Helmich, F.; Lee, C. C.; Nieuwenhuizen, M. M. L.; Gielen, J. C.; Christianen, P. C. M.; Larsen, A.; Fytas, G.; Leclère, P. E. L. G.; Schenning, A. P. H. J.; Meijer, E. W. Dilution-induced self-assembly of porphyrin aggregates: A consequence of coupled equilibria. *Angew. Chem. Int. Ed.* **2010**, *49*, 3939–3942.
3. Zhang, F.; Das, S.; Walkinshaw, A. J.; Casitas, A.; Taylor, M.; Suero, M. G.; Gaunt, M. J. Cu-Catalyzed Cascades to Carbocycles: Union of Diaryliodonium Salts with Alkenes or Alkynes Exploiting Remote Carbocations. *J. Am. Chem. Soc.* **2014**, *136*, 8851–8854.
4. Camerel, F.; Ulrich, G.; Ziessel, R. New Platforms Integrating Ethynyl-Grafted Modules for Organogels and Mesomorphic Superstructures. *Org. Lett.* **2004**, *6*, 4171–4174.
5. Rao, K. V.; Miyajima, D.; Nihonyanagi, A.; Aida, T. Thermally Bisignate Supramolecular Polymerization. *Nat. Chem.* **2017**, *9*, 1133–1139.
6. Adelizzi, B.; Filot, I. A. W.; Palmans, A. R. A.; Meijer, E. W. Unravelling the Pathway Complexity in Conformationally Flexible *N*-Centered Triarylamine Trisamides. *Chem. Eur. J.* **2017**, *23*, 6103–6110.

7. Stals, P. J. M.; Everts, J. C.; de Bruijn, R.; Filot, I. A. W.; Smulders, M. M. J.; Martín-Rapún, R.; Pidko, E. A.; de Greef, T. F. A.; Palmans, A. R. A.; Meijer, E. W. Dynamic supramolecular polymers based on benzene-1,3,5-tricarboxamides: The influence of amide connectivity on aggregate stability and amplification of chirality. *Chem. Eur. J.* **2010**, *16*, 810–821.
8. Ślęczkowski, M. L.; Mabesoone, M. F. J.; Ślęczkowski, P.; Palmans, A. R. A.; Meijer, E. W. Competition between chiral solvents and chiral monomers in the helical bias of supramolecular polymers. *Nat. Chem.* **2021**, *13*, 200–207.
9. Zhao, D.; Moore, J. S. Nucleation-elongation: A mechanism for cooperative supramolecular polymerization. *Org. Biomol. Chem.* **2003**, *1*, 3471–3491.
10. Mabesoone, M. F. J.; Markvoort, A. J.; Banno, M.; Yamaguchi, T.; Helmich, F.; Naito, Y.; Yashima, E.; Palmans, A. R. A.; Meijer, E. W. Competing Interactions in Hierarchical Porphyrin Self-Assembly Introduce Robustness in Pathway Complexity. *J. Am. Chem. Soc.* **2018**, *140*, 7810–7819.
11. Rao, K. V.; Mabesoone, M. F. J.; Miyajima, D.; Nihonyanagi, A.; Meijer, E. W.; Aida, T. Distinct Pathways in 'Thermally Bisignate Supramolecular Polymerization': Spectroscopic and Computational Studies. *J. Am. Chem. Soc.* **2020**, *142*, 598–605.
