# Supplementary material for: Simulating Assembly Landscapes for Comprehensive Understanding of Supramolecular Polymer–Solvent Systems
Source: J Am Chem Soc. 2023 Feb 9;145(7):4231–7. doi: 10.1021/jacs.2c12941 (PMC9951209; doi:10.1021/jacs.2c12941)
Supplement: Supplementary file 3 — ja2c12941_si_003.pdf [file ja2c12941_si_003.pdf]

```
% CooperativePolymerization_Fit.m

clear, close all
clc

%% Input of initial guesses
% SET UPPER AND LOWER LIMITS FOR THE SAMPLING OF ALL PARAMETERS
% Values for the Gibbs free energy and entropy of the polymerization in
kJ/mole
dGe_min = -10;
dGe_max = -40;

% Values for the entropy of the polymerization in kJ/mole
dS_min = -0.100;
dS_max = -0.150;

% Values for the Gibbs free energy and entropy of the nucleation in kJ/mole
dGn_min = -5;
dGn_max = -40;

% Polymer UV activity or absorbance
% Ep_min = 1e6;
% Ep_max = 1.2e6;
Ep_min = -6e3;
Ep_max = -1e4;

% Monomer UV activity or absorbance
Em = 0;
Constants = [Em];

% Set number of individual parameter sets that will be optimized
J = 500;

%% Generate starting parameters the thermodynamic parameters using latin
hypercube sampling
param=lhsdesign(J,4); %lhsdesign returns random values from 0 to 1 for each
parameter with one from each interval 0-1/J, 1/J-2/J,...,1-1/J-1

param(:,1) = dGe_min+(dGe_max-dGe_min).*param(:,1);
param(:,2) = dS_min+(dS_max-dS_min).*param(:,2);
param(:,3) = dGn_min+(dGn_max-dGn_min).*param(:,3);
param(:,4) = Ep_min+(Ep_max-Ep_min).*param(:,4);

%% Convert the starting parameters to the ones taken by the cost function
% Convert the Gibbs free energies to reasonable random enthalpies
param(:,1) = param(:,1) + 293*param(:,2); % Enthalpy of elongation
param(:,3) = param(:,3) + 293*param(:,2); % Enthalpy of nucleation

%% load and prepare data
% The script takes a .txt file with in the 1st column all the
% tempratures, the 2nd column the CD signal, the 3rd column the HT voltage
% of the Jasco spectrometer, the 4th column the UV signal and the 5th
% column the concentration in micromolar.
```

```
[FileName, PathName] = uigetfile({'*.txt'},'MultiSelect','off');
if iscell(FileName)
    FileName = cell2mat(FileName);
end

dat=load(strcat(PathName,FileName));
dat = dat([1:2:end],:); % Take every 2nd data point to reduce the number of
calculations

% Define the format of the data matrix
T = dat(:,1);  % Temperatures
CD = dat(:,2); % CD signal
UV = dat(:,3); % UV signal
c = dat(:,4); % Concentration TAA in uM
normCD = CD./c; % Calculate the CD, corrected for concentration. If fitting
UV, change this line!

% If the input data is still in degrees Celsius, convert to Kelvin
if min(T)<100
    T = T+273.15;
end

%% Correct for baseline drift
[samples,ind_samples,~] = unique([c],'rows','stable');
samplerange = zeros(size(ind_samples,1),2);
samplerange(1,:) = [1,ind_samples(2)-1];
for i = 2:length(ind_samples)-1
    samplerange(i,:) = [ind_samples(i),ind_samples(i+1)-1];
end
samplerange(size(ind_samples,1),:) =
[ind_samples(size(ind_samples,1)),length(c)];

% Correct CD to baseline above Te based on highest 10 datapoints
for i = 1:length(samples)
    av_highest10C = sum(normCD(samplerange(i,1):samplerange(i,1)+9,1))./10;

normCD(samplerange(i,1):samplerange(i,2))=normCD(samplerange(i,1):samplerange
(i,2))-av_highest10C;
end

% % Correct UV for baseline and baseline drift
% for i = 1:length(samples)
%     av_highest10C = sum(normCD(samplerange(i,1):samplerange(i,1)+9,1))./10;
%
normUV(samplerange(i,1):samplerange(i,2))=UV(samplerange(i,1):samplerange(i,2
))-UV(samplerange(i,1));
%     C50 = find(T(samplerange(i,1):samplerange(i,2)) >= T(samplerange(i,1))-
10);
%     C50i(i) = samplerange(i,1)-1+max(C50);
%     dx = (normUV(samplerange(i,1))-normUV(C50i(i)))./(T(samplerange(i,1))-
T(C50i(i)));
%     for j = samplerange(i,1):samplerange(i,2)
%     normUV(j) = dx.*(T(samplerange(i,1))-T(j))+normUV(j);
%     end
```

```
%
normUV(samplerange(i,1):samplerange(i,2))=normUV(samplerange(i,1):samplerange
(i,2))./samples(i);
% end

%% Prepare for fitting
% Define an initial error, used for checking. This one is chosen large on
% purpose, so that the first fit will be the first minimum.
r_check=1e99;

check=[];
allresnorm=[];
AllPar_Fin = [];
AllJacob = [];
AllRes = [];

%% Define options for optimalization
max_iter = 10; % THe maximum iterations that is passed to the interrupt
function

fprintf('Progress: %g %% \n\n', 0/J*100);

j=1; % First iteration
Freport=[]; %in this matrix the failed and succesful parametersets are saved
Sreport=[]; %in this matrix the succesfull initial parametersets are saved

% Boundaries. Because we use the Levenberg-Marquardt algorithm, no
% boundaries should be passed to lsqnonlin.
lb = [];
ub = [];

%% Call lsqnnonlin
while j<=J %for all parameter sets
     %load parameter values for current parameter set j
%     outputFun=
@(x,optimValues,state,varargin)HS_interuptFun(x,optimValues,state,varargin,r_
check,max_iter);
    options=optimset('MaxIter',250,'Display','Iter','MaxFunEvals',4000,...
            'TolX',1e-25,'TolFun',1e-
25,'UseParallel',1,'Algorithm','levenberg-marquardt','useParallel',true); %
If the parallel toolbox is not installed, set useParallel to 'false'

    % Use a try-catch block. Some starting values do not give solutions and
lead to an error. The try-catch prevents crashing of the script.
    try
        % Optimize the j-th parameter set using lsqnonlin.
        [par_fin,resnorm,residual,exitflag,output,lambda,Jacobian]=...

lsqnonlin(@CooperativePolymerization_Cost,param(j,:),lb,ub,options, T,
normCD, c, Constants);

        %if the optimized parameters give a lower norm of the residual than
        %the previous best fit, save the newly optimized parameters and
        %some other data
        if resnorm<r_check
```

```
            BestPar_Fin = par_fin;
            r_check=resnorm;
            BestIndex = j;
            BestJacob = Jacobian;
            BestRes = residual;
            exitMes = output;
        end

        % Save some output of all optimizations
        %allresnorm is a 4 x J matrix with the first column values of J
(number of parameterset) and the second column containing the coresponding
resnorm
        allresnorm=[allresnorm; resnorm]; %resnorm is the sum of all squared
residuals
        if ~isrow(par_fin)
            par_fin = par_fin';
        end
        AllPar_Fin = [AllPar_Fin; par_fin];
        AllRes = [AllRes;residual'];

        % Progress indicator
        fprintf('Progress: %g %% \n Resnorm: %5f\n', j/J*100,r_check);

        %Go to next parameterset
        j=j+1;

    catch
        % If a parameter set failed to give a solution, try a new random
        % parameter set
        newparam=lhsdesign(1,4); %lhsdesign returns random values from 0 to 1
for each parameter with one from each interval 0-1/J, 1/J-2/J,...,1-1/J-1

        newparam(:,1) = dGe_min+(dGe_max-dGe_min).*newparam(:,1);
        newparam(:,2) = dS_min+(dS_max-dS_min).*newparam(:,2);
        newparam(:,3) = dGn_min+(dGn_max-dGn_min).*newparam(:,3);
        newparam(:,4) = Ep_min+(Ep_max-Ep_min).*newparam(:,4);

        %% Convert the starting newparameters to the ones taken by the cost
function
        newparam(:,1) = newparam(:,1) + 293*newparam(:,2);
        newparam(:,3) = newparam(:,3) + 293*newparam(:,2);

        % Convert nucleation penalty to entropy of nucleation
        param(j,:) = newparam;
        display('newpar')
    end
end

%% Analysis of the fit results
% Check if there are multiple iterations with similar ResNorm but different
% Par_Fin
BestFitIndices = find(allresnorm==r_check);

if length(BestFitIndices)>1
    all = AllPar_Fin(BestFitIndices,:);
```

```
    Diff = all - ones(size(all,1),1)*BestPar_Fin;
    if sum(sum(Diff))~=0
        warning('Multiple minima are detected') % If mutiple parameter sets
give identical fits, give a warning.
    end
end

% Extract best fit parameters
He_fit = BestPar_Fin(1);
S_fit = BestPar_Fin(2);
Hn_fit = BestPar_Fin(3);
Ep_fit = BestPar_Fin(4);

% Also find the fits that are within 5% of the best fit
indices = find(allresnorm<= 1.05*r_check);
GoodFits = AllPar_Fin(indices,:);
GoodFitsLog = sign(GoodFits).*log10(abs(GoodFits)); % Convert the fits of the
other, almost as good fits, to their logarithm for better visualization. This
is because the values of dH and dS differ so much

%% Output
multipleFit = 'no';
[samples,ind_samples,~] = unique([c],'rows','stable');
samplerange = zeros(size(ind_samples,1),2);
samplerange(1,:) = [1,ind_samples(2)-1];
for i = 2:length(ind_samples)-1
    samplerange(i,:) = [ind_samples(i),ind_samples(i+1)-1];
end
samplerange(size(ind_samples,1),:) =
[ind_samples(size(ind_samples,1)),length(c)];

col = [0.03,0.62,0.00;... %1
% 0.02,0.48,0.14;... %2
0.02,0.45,0.17;... %3
% 0.02,0.41,0.21;... %4
0.02,0.40,0.23;... %5
% 0.02,0.36,0.27;... %6
0.01,0.25,0.38;... %7
% 0.01,0.17,0.46;... %8
0.00,0.08,0.55;... %9
% 0.00,0.05,0.59;... %10
0.00,0.02,0.63]; %11

Trange = linspace(min(T),max(T),50)';
onesmatrix = ones(size(Trange));
matrix_CDcalc = [Trange-273.15];

for r = 1:length(samples)
figure(1)
scatter(T(samplerange(r,1):samplerange(r,2))-
273.15,normCD(samplerange(r,1):samplerange(r,2)),75,'filled','MarkerFaceColor
',col(r,:),'MarkerFaceAlpha',1/10)
hold on
[M,P] = CooperativePolymerization_Sim(BestPar_Fin, Trange,
samples(r).*onesmatrix);
CDcalc = (Em.*M+Ep_fit.*P)./samples(r);
```

```
plot(Trange-273.15,CDcalc,'Color',col(r,:),'LineWidth',1.5)
hold on
matrix_CDcalc = [matrix_CDcalc,CDcalc];
title(BestPar_Fin)
end
figure(1)

% for r = 1:length(samples)
% figure(1)
% scatter(T(samplerange(r,1):samplerange(r,2))-
273.15,normUV(samplerange(r,1):samplerange(r,2)),75,'filled','MarkerFaceColor
',col(r,:),'MarkerFaceAlpha',1/10)
% hold on
% [M,P] = CooperativePolymerization_Sim(BestPar_Fin, Trange,
samples(r).*onesmatrix);
% UVcalc = (Em.*M+Ep_fit.*P)./samples(r);
% plot(Trange-273.15,UVcalc,'Color',col(r,:),'LineWidth',1.5)
% hold on
% title(BestPar_Fin)
% end
% figure(1)

% Output some data to the command window
fprintf('____________________________________________________________________
_____________\n');
fprintf('Elongation enthalpy He = %d \n',He_fit);
fprintf('Elongation entropy Se = %d \n',S_fit);
fprintf('\t\tdGe = %d \n',[He_fit-293*S_fit]);
fprintf('____________________________________________________________________
_____________\n');
fprintf('Nucleation enthalpy Hn =%d \n',Hn_fit);
fprintf('\t\tdGn = %d \n',[Hn_fit-293*S_fit]);
fprintf('____________________________________________________________________
_____________\n');
fprintf('Molar coextinction =%d \n',Ep_fit);
fprintf('____________________________________________________________________
_____________\n');
```

```
% CooperativePolymerization_Cost.m

% This cost function is used in the fitting routine to define the quality
% of the current fit. This function outputs a vector that contains the
% difference between the experimental data and calculated signal for
% every datapoint.

function [difference] = CooperativePolymerization_Cost(par, T, signal, C,
Constants)
% Cost function for the fitting routine of cooperative polymerizations
Em = Constants(1);
Ep = par(4);

% Calculate the monomer distribution over the various species
[M, P] = CooperativePolymerization_Sim(par,T,C);

% % Convert this distribution to a CD signal
Signal_calc = (M.*Em + Ep.*P)./C;

% Calculate the cost vector
difference = Signal_calc-signal;
end
```

```matlab
% CooperativePolymerization_Sim.m

% This sim function is used to simulate the cooperative polymerization at
% different conditions. The function takes the temperature and
% concentrations from the experimental data and calculates the binding
% constants based on these conditions. As output, the function creates a
% matrix with the free monomer in the 1st column and the polymerized
% concentration of monomers in the 2nd column.

function [M,P] = CooperativePolymerization_Sim(par, T, c)

if isrow(T)
    T = T';
end

% Extract parameters
He = par(1);
Se = par(2);
Hn = par(3);

R = 0.0083145; % Gas constant

% Generate empty array to store the free monomer (M) concentrations
M = zeros(length(T),1);

Kn = exp(-(Hn-T.*Se)./(R.*T));
Ke = exp(-(He-T.*Se)./(R.*T));
sigma = Kn./Ke;
for i = 1:length(T)
    % Calculate the free monomer concentration for every datapoint
    M(i) = CooperativePolymerization_Solver(c(i), sigma(i), Ke(i));
end
% Calculate the polymerized concentration of monomers based on the free
% monomer concentration
P = sigma.*M./(1-Ke.*M).^2-sigma.*M;
end
```

```
% CooperativePolymerization_Solver.m

% This solver function calculates the free monomer concentration for a
% cooperative polymerization based on the mass-balance equation.
% The input parameters required are the concentration, the cooperativity
% parameter at this temperature and the binding constant for elongation
% at this temperature. The output is the free  monomer concentration at
% these conditions.

function [Monomer] = CooperativePolymerization_Solver(c,sigma,Ke)

% Estimate the range in which the solution has to be
xguess = [0,1/Ke-1e-30]; % Make sure that the upper limit is just inside the
[0 1/Ke] limit, so you don't reach the asymptote

errL = FreeMonomerCalculator(xguess(1),c,Ke,sigma); % Error on left extreme
errR = FreeMonomerCalculator(xguess(2),c,Ke,sigma); % Error on right extreme.
Should have opposite sign of errL.
count = 0; % Create a counter that will exit after 100 times, to prevent
infinite loops
while (errL < -1e-15) & ((xguess(2)-xguess(1))>1e-14) & count <1e2
    xM = (xguess(1)+xguess(2))/2; % Take a value halfway
    errM = FreeMonomerCalculator(xM,c,Ke,sigma); % Calculate the error
    if errM < 0 % Assign new boundaries of the relevant region
        xguess(1) = xM;
        errL = errM;
    else
        xguess(2) = xM;
        errR = errM;
    end
    count = count + 1;
end
Monomer = xguess(1);
end

function y = FreeMonomerCalculator(M,Mtot,Ke,sigma)
% Calculate the difference between the right hand side and left hand side of
the mass balance equation
y = (1-sigma).*M + sigma.*M./(1-Ke.*M).^2 - Mtot;
end
```
