# Supplementary material for: Simulating Assembly Landscapes for Comprehensive Understanding of Supramolecular Polymer–Solvent Systems
Source: J Am Chem Soc. 2023 Feb 9;145(7):4231–7. doi: 10.1021/jacs.2c12941 (PMC9951209; doi:10.1021/jacs.2c12941)
Supplement: Supplementary file 4 — ja2c12941_si_004.pdf [file ja2c12941_si_004.pdf]

```matlab
% Assembly_Landscape_Runner.m

clear all
close all

Eq_S = 4280; % S-Por1Zn
% Eq_S = 13700; % S-Por2Zn
% Eq_S = 2140; % S-TPA
% Eq_S = 1000; % S-Por2Cu

Mtots = logspace(-6,-4,200);
Ts = linspace(253,383,200);

%% Thermodynamic Parameters
par = [-35.6,-0.0896,-9.7,-0.248,2000,-70,-0.0976,20]; % S-Por1Zn
% par = [-43.7,-0.1101,-37.9,-0.103,0,-107.1,-0.2092,3.5]; % S-Por2Zn
% par = [-51.6,-0.1432,-9.7,-0.248,0,-91.8,-0.1942,8.7]; % S-TPA
% par = [-27.9,-0.0508,-37.9,-0.103,0,-73.2,-0.0798,0]; % S-Por2Cu

Hcom = par(1);
Scom = par(2);
Hclus = par(3);
Sclus = par(4);
m = par(5);
Hpol = par(6);
Spol = par(7);
NP = par(8);

gasconstant=0.008314472; % in: kJ/mol K

number_cluster = 4; % S-Por1Zn
% number_cluster = 8; % S-Por2Cu, S-Por2Zn
% number_cluster = 3; % S-TPA

%% Calculation
for i = 1:length(Mtots)
    Mtot = Mtots(i);
    Stots(i) = Eq_S.*Mtot;
    Stot = Stots(i);
    f = (Eq_S*Mtot*46.07)/(10*0.789)/100;

    for j = 1:length(Ts)
        T = Ts(j);
        RT = gasconstant*T;

        Ke = exp(-(Hpol-T*Spol+m*f)/RT);
        sigma = exp(-NP/RT);
        Kcom = exp(-(Hcom-T*Scom)/RT);
        Kclus = exp(-(Hclus-T*Sclus)/RT);

        par = [Ke,sigma,Kcom,Kclus,number_cluster];
        [M(i,j),H(i,j),SeqM(i,j),SS(i,j)] =
PolymerSolvent_Solver(par,Mtot,Stot); % Solve the mass balance
        Monomer(i,j) = M(i,j)/Mtot;
```

```
        Polymerized(i,j) = H(i,j)/Mtot;
        Sequestrated(i,j) = SeqM(i,j)/Mtot;
        Clustered(i,j) = SS(i,j)/Stot;
    end
end

ind = 1;
for j = 1:length(Mtots)
    for i = 1:length(Ts)
        if Polymerized(j,i) == 0.5
            polborders(ind,:) = [Ts(i),Mtots(j)];
            ind = ind+1;
        elseif Polymerized(j,i) > 0.48 && Polymerized(j,i) < 0.52
            polborders(ind,:) = [Ts(i),Mtots(j)];
            ind = ind+1;
        end
        if Sequestrated(j,i) == 0.5
            seqborders(ind,:) = [Ts(i),Mtots(j)];
            ind = ind+1;
        elseif Sequestrated(j,i) > 0.48 && Sequestrated(j,i) < 0.52
            seqborders(ind,:) = [Ts(i),Mtots(j)];
            ind = ind+1;
        end
        if Polymerized(j,i) < 0.5 && Sequestrated(j,i) < 0.5 && Monomer(j,i)
< 0.5
            borders(ind,:) = [Ts(i),Mtots(j)];
            ind = ind+1;
        end
    end
end

output = [Mtots',Polymerized];

allborders = [polborders;seqborders;borders];

figure
fig = pcolor(Ts-273.15,Mtots,Polymerized)
fig.EdgeColor = 'none';
hold on
scatter(allborders(:,1)-
273.15,allborders(:,2),5,'MarkerFaceColor',[1,0,0],'MarkerEdgeColor',[1,0,0])
hold on
set(gca, 'YScale', 'log');
xlabel('Temperature (C)')
ylabel('Monomer concentration (M)')
title('Polymerized')
colorbar
```

```
% PolymerSolvent_Fit.m

clear, close all
clc

%% Input of initial guesses
OutputFollower = 0  ;
%[-35.5825768802910,-0.0896121051207454,-9.72333718936685,-
0.248047653609938,222944.445285349,2000,-70.0000000000000,-
0.0976499532635236,39.9999547423438]
% SET UPPER AND LOWER LIMITS FOR THE SAMPLING OF ALL PARAMETERS
% 1.
% Values for the Gibbs free energy and entropy of the polymerization at high
temperature in kJ/mole
dGcom_min = -5;
dGcom_max = -25;

dScom_min = -0.050;
dScom_max = -0.150;

% dGclus_min = -1;
% dGclus_max = -5;
%
% dSclus_min = -0.150;
% dSclus_max = -0.300;

Ep_min = -6e7;
Ep_max = -6.5e7;

% m_min = 0;
% m_max = 1000;
%
dGpol_min = -35;
dGpol_max = -50;

dSpol_min = -0.050;
dSpol_max = -0.150;

NP_min = 10;
NP_max = 30;

c = 50e-6;

% Set number of individual parameter sets that will be optimized
J = 500;

%% Generate starting parameters the thermodynamic parameters using latin
hypercube sampling
param=lhsdesign(J,6); %lhsdesign returns random values from 0 to 1 for each
parameter with one from each interval 0-1/J, 1/J-2/J,...,1-1/J-1

param(:,1) = dGcom_min+(dGcom_max-dGcom_min).*param(:,1);
param(:,2) = dScom_min+(dScom_max-dScom_min).*param(:,2);
% param(:,3) = dGclus_min+(dGclus_max-dGclus_min).*param(:,3);
```

```
% param(:,4) = dSclus_min+(dSclus_max-dSclus_min).*param(:,4);
param(:,6) = Ep_min+(Ep_max-Ep_min).*param(:,6);
% param(:,6) = m_min+(m_max-m_min).*param(:,6);
param(:,3) = dGpol_min+(dGpol_max-dGpol_min).*param(:,3);
param(:,4) = dSpol_min+(dSpol_max-dSpol_min).*param(:,4);
param(:,5) = NP_min+(NP_max-NP_min).*param(:,5);

%% Convert the starting parameters to the ones taken by the cost function
% Convert the Gibbs free energies to reasonable random enthalpies
param(:,1) = param(:,1) + 293*param(:,2); % Enthalpy of elongation
param(:,3) = param(:,3) + 293*param(:,4); % Enthalpy of elongation
% param(:,7) = param(:,7) + 293*param(:,8); % Enthalpy of elongation

%% load and prepare data
% The script takes a .txt file with in the 1st column all the
% tempratures, the 2nd column the CD signal, the 3rd column the HT voltage
% of the Jasco spectrometer, the 4th column the UV signal and the 7th
% column the normalized CD, the 8th column the concentration in micromolar.

[FileName, PathName] = uigetfile({'*.txt'},'MultiSelect','off');
if iscell(FileName)
    FileName = cell2mat(FileName);
end

dat=load(strcat(PathName,FileName));
dat = dat([1:2:end],:);

T = dat(:,1);  % Temperatures
CD = dat(:,2);
UV = dat(:,3); % UV signal
c = dat(:,4); % HT signal
normCD = CD./c;

% indices = find(c==10e-6);
% normUV(indices) = normUV(indices)*3;

% If the input data is still in degrees Celsius, convert to Kelvin
if min(T)<100
    T = T+273.15;
end

%% Baseline correction
% [samples,ind_samples,~] = unique([c],'rows','stable');
% samplerange = zeros(size(ind_samples,1),2);
% samplerange(1,:) = [1,ind_samples(2)-1];
% for i = 2:length(ind_samples)-1
%     samplerange(i,:) = [ind_samples(i),ind_samples(i+1)-1];
% end
% samplerange(size(ind_samples,1),:) =
[ind_samples(size(ind_samples,1)),length(c)];
%
% % Correct CD to baseline above Te based on highest 10 datapoints
% for i = 1:length(samples)
%     av_highest10C = sum(normCD(samplerange(i,1):samplerange(i,1)+9,1))./10;
```

```
%
normCD(samplerange(i,1):samplerange(i,2))=normCD(samplerange(i,1):samplerange
(i,2))-av_highest10C;
% end

%% Prepare for fitting
% Define an initial error, used for checking. This one is chosen large on
% purpose, so that the first fit will be the first minimum.
r_check=1e99;

check=[];
allresnorm=[];
AllPar_Fin = [];
AllJacob = [];
AllRes = [];

%% Define options for optimalization
max_iter = 10; % THe maximum iterations that is passed to the interrupt
function

fprintf('Progress: %g %% \n\n', 0/J*100);

j=1; % First iteration
Freport=[]; %in this matrix the failed and succesful parametersets are saved
Sreport=[]; %in this matrix the succesfull initial parametersets are saved

% Boundaries. Because we use the Levenberg-Marquardt algorithm, no
% boundaries should be passed to lsqnonlin.
lb = [];
ub = [];

%% Call lsqnnonlin
while j<=J %for all parameter sets
     %load parameter values for current parameter set j
    options=optimset('MaxIter',250,'Display','Iter','MaxFunEvals',4000,...
            'TolX',1e-25,'TolFun',1e-
25,'UseParallel',1,'Algorithm','levenberg-marquardt','useParallel',true); %
If the parallel toolbox is not installed, set useParallel to 'false'

    % Use a try-catch block. Some starting values do not give solutions and
lead to an error. The try-catch prevents crashing of the script.
    try
        % Optimize the j-th parameter set using lsqnonlin.
        [par_fin,resnorm,residual,exitflag,output,lambda,Jacobian]=...
            lsqnonlin(@PolymerSolvent_Cost,param(j,:),lb,ub,options, T,
normCD, c);

        %if the optimized parameters give a lower norm of the residual than
        %the previous best fit, save the newly optimized parameters and
        %some other data
        if resnorm<r_check
            BestPar_Fin = par_fin;
            r_check=resnorm;
            BestIndex = j;
            BestJacob = Jacobian;
```

```
            BestRes = residual;
            exitMes = output;
        end

        % Save some output of all optimizations
        %allresnorm is a 4 x J matrix with the first column values of J
(number of parameterset) and the second column containing the coresponding
resnorm
        allresnorm=[allresnorm; resnorm]; %resnorm is the sum of all squared
residuals
        if ~isrow(par_fin)
            par_fin = par_fin';
        end
        AllPar_Fin = [AllPar_Fin; par_fin];
        AllRes = [AllRes;residual'];

        % Progress indicator
        fprintf('Progress: %g %% \n Resnorm: %5f\n', j/J*100,r_check);

        %Go to next parameterset
        j=j+1;

    catch
        % If a parameter set failed to give a solution, try a new random
        % parameter set
        newparam=lhsdesign(1,6); %lhsdesign returns random values from 0 to 1
for each parameter with one from each interval 0-1/J, 1/J-2/J,...,1-1/J-1
        newparam(:,1) = dGcom_min+(dGcom_max-dGcom_min).*newparam(:,1);
        newparam(:,2) = dScom_min+(dScom_max-dScom_min).*newparam(:,2);
%         newparam(:,3) = dGclus_min+(dGclus_max-dGclus_min).*newparam(:,3);
%         newparam(:,4) = dSclus_min+(dSclus_max-dSclus_min).*newparam(:,4);
        newparam(:,6) = Ep_min+(Ep_max-Ep_min).*newparam(:,6);
%         newparam(:,6) = m_min+(m_max-m_min).*newparam(:,6);
        newparam(:,3) = dGpol_min+(dGpol_max-dGpol_min).*newparam(:,3);
        newparam(:,4) = dSpol_min+(dSpol_max-dSpol_min).*newparam(:,4);
        newparam(:,5) = NP_min+(NP_max-NP_min).*newparam(:,5);

        %% Convert the starting newparameters to the ones taken by the cost
function
        newparam(:,1) = newparam(:,1) + 293*newparam(:,2);
        newparam(:,3) = newparam(:,3) + 293*newparam(:,4);
%         newparam(:,7) = newparam(:,7) + 293*newparam(:,8);

        % Convert nucleation penalty to entropy of nucleation
        param(j,:) = newparam;
        display('newpar')
    end
end

%% Analysis of the fit results
% Check if there are multiple iterations with similar ResNorm but different
% Par_Fin
BestFitIndices = find(allresnorm==r_check);

if length(BestFitIndices)>1
```

```
    all = AllPar_Fin(BestFitIndices,:);
    Diff = all - ones(size(all,1),1)*BestPar_Fin;
    if sum(sum(Diff))~=0
        warning('Multiple minima are detected') % If mutiple parameter sets
give identical fits, give a warning.
    end
end

% Extract best fit parameters
Hcom_fit = BestPar_Fin(1);
Scom_fit = BestPar_Fin(2);
% Hclus_fit = BestPar_Fin(3);
% Sclus_fit = BestPar_Fin(4);
% Ep_fit = 1.0172e+06; % S-TPA
Ep_fit = BestPar_Fin(6);
% m_fit = BestPar_Fin(6);
Hpol_fit = BestPar_Fin(3);
Spol_fit = BestPar_Fin(4);
NP_fit = BestPar_Fin(5);

% Also find the fits that are within 5% of the best fit
indices = find(allresnorm<= 1.05*r_check);
GoodFits = AllPar_Fin(indices,:);
GoodFitsLog = sign(GoodFits).*log10(abs(GoodFits)); % Convert the fits of the
other, almost as good fits, to their logarithm for better visualization. This
is because the values of dH and dS differ so much

%% Output
multipleFit = 'no';
[samples,ind_samples,~] = unique([c],'rows','stable');
samplerange = zeros(size(ind_samples,1),2);
samplerange(1,:) = [1,ind_samples(2)-1];
for i = 2:length(ind_samples)-1
    samplerange(i,:) = [ind_samples(i),ind_samples(i+1)-1];
end
samplerange(size(ind_samples,1),:) =
[ind_samples(size(ind_samples,1)),length(c)];

col = [0.03,0.62,0.00;... %1
% 0.02,0.48,0.14;... %2
% 0.02,0.45,0.17;... %3
0.02,0.41,0.21;... %4
% 0.02,0.40,0.23;... %5
0.02,0.36,0.27;... %6
% 0.01,0.25,0.38;... %7
0.01,0.17,0.46;... %8
% 0.00,0.08,0.55;... %9
% 0.00,0.05,0.59;...
0.00,0.02,0.63]; %10

Trange = linspace(min(T),max(T),100)';
onesmatrix = ones(size(Trange));
matrix_CDcalc = [Trange-273.15];
matrix_normCD = [];
for r = 1:length(samples)
figure(1)
```

```
scatter(T(samplerange(r,1):samplerange(r,2))-
273.15,normCD(samplerange(r,1):samplerange(r,2)),75,'filled','MarkerFaceColor
',col(r,:),'MarkerFaceAlpha',1/10)
hold on
[M,H,SeqM,SS] = PolymerSolvent_Sim(BestPar_Fin, Trange,
samples(r).*onesmatrix);
CDcalc = (Ep_fit.*H)./samples(r);
matrix_CDcalc = [matrix_CDcalc,CDcalc];
matrix_normCD = [matrix_normCD,T(samplerange(r,1):samplerange(r,2))-
273.15,normCD(samplerange(r,1):samplerange(r,2))];
plot(Trange-273.15,CDcalc,'Color',col(r,:),'LineWidth',1.5)
hold on
% figure(2)
%
scatter(T(samplerange(r,1):samplerange(r,2)),normCD(samplerange(r,1):samplera
nge(r,2)),75,'filled','MarkerFaceColor',col(r,:),'MarkerFaceAlpha',1/10)
% hold on
% matrix_UVcalc = [matrix_UVcalc,UVcalc];
end
figure(1)
title(BestPar_Fin)
% xlim([10 110])
% figure(2)
% xlim([10 110])

% Output some data to the command window
%
fprintf('_____________________________________________________________________________
_____________\n');
% fprintf('Elongation enthalpy He = %d \n',He_fit);
% fprintf('Elongation entropy Se = %d \n',S_fit);
% fprintf('\t\tdGe = %d \n',[He_fit-293*S_fit]);
%
fprintf('_____________________________________________________________________________
_____________\n');
% fprintf('Nucleation enthalpy Hn =%d \n',Hn_fit);
% fprintf('\t\tdGn = %d \n',[Hn_fit-293*S_fit]);
%
fprintf('_____________________________________________________________________________
_____________\n');
% fprintf('Molar coextinction =%d \n',Ep_fit);
%
fprintf('_____________________________________________________________________________
_____________\n');


% FileID = fopen(strcat(PathName,FileName(1:end-4),'_param.txt'),'w');
% txt = sprintf('He = %5e\r\nS=%5e\r\nHn = %5e\r\n\nResnorm = %5e\r\n\nFound
multiple good fits: %s',...
%     He_fit,S_fit,Hn_fit,r_check,multipleFit);
% fprintf(FileID,'%s',txt);
```

```
% PolymerSolvent_Cost.m

% Cost function for the fitting routine. This function outputs a vector
% that contains the difference between the experimental data and calculated
% signal for every datapoint.
%
function [cost] = PolymerSolvent_Cost(par, T, signal, C)
% Cost function for the fitting routine of the thermally bisignate pol
Ep = par(6);
% Ep = 1.0172e+06; % S-TPA

% Calculate the monomer distribution over the various species
[M,H,SeqM,SS] = PolymerSolvent_Sim(par,T,C);

% Convert this distribution to a CD signal
Signal_calc = (Ep.*H)./C;

% Calculate the cost vector
cost = Signal_calc-signal;

% indices = find(C==2e-6);
% cost(indices) = cost(indices);
%
% indices = find(C==5e-6);
% cost(indices) = cost(indices);
%
% indices = find(C==10e-6);
% cost(indices) = 2.*cost(indices);
end
```

```
% PolymerSolvent_Sim.m

function [M,H,SeqM,SS] = PolymerSolvent_Sim(par, T, c)

if isrow(T)
    T = T';
end

% A cooperative polymerization. The mass balance equations in this script
% consider a cooperative polymerization with nucleus size 2.

% Extract parameters
Hcom = par(1);
Scom = par(2);
% Hclus = par(3);
% Sclus = par(4);
Hclus = -9.7; % MCH
Sclus = -0.248; % MCH
% Hclus = -37.9; % MCH/CHCl3 98/2
% Sclus = -0.103; % MCH/CHCl3 98/2
% m = par(6);
m = 0;
Hpol = par(3);
Spol = par(4);
NP = par(5);
% Hpol = -91.8388; % S-TPA
% Spol = -0.194189; % S-TPA
% NP = -Hpol-83.11; % S-TPA

Eq_S = 13700;
number_cluster = 8;

gasconstant = 0.0083145; % Gas constant

% Generate empty arrays to store the free monomer (M) concentrations
M = zeros(length(T),1);
H = zeros(length(T),1);
SeqM = zeros(length(T),1);
SS = zeros(length(T),1);

for i = 1:length(T)
    Ti = T(i);
    RT = gasconstant*Ti;
    f = (Eq_S*c(i)*46.07)/(10*0.789)/100;

    Ke = exp(-(Hpol-Ti*Spol+m*f)/RT);
    sigma = exp(-NP/RT);
    Kcom = exp(-(Hcom-Ti.*Scom)./(RT));
    Kclus = exp(-(Hclus-Ti.*Sclus)./(RT));

    parsolver = [Ke,sigma,Kcom,Kclus,number_cluster];
    % Calculate the free monomer and alcohol concentration for every
temperature
```

```
    [M(i),H(i),SeqM(i),SS(i)] = PolymerSolvent_Solver(parsolver, c(i), 
Eq_S.*c(i));
end

end
```

% PolymerSolvent_Solver.m

```
function [M,H,SeqM,SS] = PolymerSolvent_Solver(par,Mtot,Stot)
Ke = par(1);
sigma = par(2);
Ksm = par(3);
Kss = par(4);
numbercluster = par(5);

% Set initial errors equal to function values at origin.
errM = M_calculator(0,Mtot,0,par);
errS = S_calculator(0,Stot,0,par);

% Set an initial starting point
S_test = 0;
M_test = 0;

% Set the reference point very far. This is just to enter the while loop
S_test_old = 1e99;
M_test_old = 1e99;

% A certain error on the solution has to be tolerated, since we are
% solving the equations numerically. In the case of a 50 uM solution, a tol
% of 1e-10 allows a maximum error of 20 ppm.
tol = 1e-10;

while ~all([abs(errM)<tol, abs(errS)<tol]) && ~all([abs(M_test-
M_test_old)<tol, abs(S_test-S_test_old)<tol])
    % Define the search boundaries
    M_guess = [0, min(1/Ke,Mtot)-1e-30];
    S_guess = [0, min(1/Kss,Stot)-1e-30];

    % Calculate the error in monomer M
    errM_L = M_calculator(M_guess(1),Mtot,S_test,par);
    errM_R = M_calculator(M_guess(2),Mtot,S_test,par);

    % Save the solution from the previous iteration
    M_test_old = M_test;
    S_test_old = S_test;

    count = 0;
    % Do the binary search for monomer M
    while any([errM_L>tol, ((M_guess(2)-M_guess(1))>1e-19)])
        xM = (M_guess(1) + M_guess(2))/2;
        errM = M_calculator(xM,Mtot,S_test,par);
        if errM > 0
            M_guess(1) = xM;
            errM_L = errM;
        else
            M_guess(2) = xM;
            errM_R = errM;
        end
        count = count + 1;
```

```
        if count > 10e6 % Have a limit in the number of iterations to prevent
an infinite loop
            error('Counter overflow for M') % No solution can be found.
Usually no problem. A new parameter set will be optimized
            return
        end
    end
    % Save the solution
    M_test = M_guess(1);
    count = 0; % Reset the counter

    % Calculate the error in additive S
    errS_L = S_calculator(S_guess(1),Stot,M_test,par);
    errS_R = S_calculator(S_guess(2),Stot,M_test,par);

    % Do the binary search for additive S
    while any([errS_L>1000*tol, ((S_guess(2)-S_guess(1))>1e-15)])
        xS = (S_guess(1) + S_guess(2))/2;
        errS = S_calculator(xS,Stot,M_test,par);
        if errS > 0
            S_guess(1) = xS;
            errS_L = errS;
        else
            S_guess(2) = xS;
            errS_R = errS;
        end
        count = count + 1;
        if count > 10e6
            display('Counter overflow for S')
            return
        end
    end
    % Save the solution
    S_test = S_guess(1);

    % Calculate the errors at the current test coordinates
    errM = M_calculator(M_test,Mtot,S_test,par);
    errS = S_calculator(S_test,Stot,M_test,par);
end
M = M_test;
S = S_test;
H = sigma.*M./(1-Ke.*M).^2-sigma.*M;
SeqM = 0;
for k = 1:numbercluster
    SeqM = SeqM+M.*(Ksm.*S).^k;
end
SS = S./(1-Kss.*S).^2-S;
end

function y = M_calculator(M,Mtot,S,par)
Ke = par(1);
sigma = par(2);
Ksm = par(3);
numbercluster = par(5);

H = sigma.*M./(1-Ke.*M).^2-sigma.*M;
```

```
SM = 0;
for k = 1:numbercluster
    SM = SM+M.*(Ksm.*S).^k;
end

y = Mtot - (M+H+SM);
end

function y = S_calculator(S,Stot,M,par)
Ksm = par(3);
Kss = par(4);
numbercluster = par(5);

SS = S./(1-Kss.*S).^2-S;

SM = 0;
for k = 1:numbercluster
    SM = SM+k.*M.*(Ksm.*S).^k;
end

y = Stot - (S+SS+SM);
end
```
